# FE65 Binds Teashirt, Inhibiting Expression of the Primate-Specific Caspase-4

**DOI:** 10.1371/journal.pone.0005071

**Published:** 2009-04-03

**Authors:** Yuji Kajiwara, Afia Akram, Pavel Katsel, Vahram Haroutunian, James Schmeidler, Gary Beecham, Jonathan L. Haines, Margaret A. Pericak-Vance, Joseph D. Buxbaum

**Affiliations:** 1 Laboratory of Molecular Neuropsychiatry, Mount Sinai School of Medicine, New York, New York, United States of America; 2 Department of Psychiatry, Mount Sinai School of Medicine, New York, New York, United States of America; 3 Department of Neuroscience, Mount Sinai School of Medicine, New York, New York, United States of America; 4 Department of Genetics and Genomic Sciences, Mount Sinai School of Medicine, New York, New York, United States of America; 5 Miami Institute for Human Genomics, University of Miami, Miller School of Medicine, Miami, Florida, United States of America; 6 Center for Human Genetics Research, Vanderbilt University, Nashville, Tennessee, United States of America; Mental Health Research Institute of Victoria, Australia

## Abstract

The Alzheimer disease (AD) amyloid protein precursor (APP) can bind the FE65 adaptor protein and this complex can regulate gene expression. We carried out yeast two-hybrid studies with a PTB domain of FE65, focusing on those genes that might be involved in nuclear signaling, and identified and validated Teashirt proteins as FE65 interacting proteins in neurons. Using reporter systems, we observed that FE65 could simultaneously recruit SET, a component of the inhibitor of acetyl transferase, and Teashirt, which in turn recruited histone deacetylases, to produce a powerful gene-silencing complex. We screened stable cell lines with a macroarray focusing on AD-related genes and identified *CASP4*, encoding caspase-4, as a target of this silencing complex. Chromatin immunoprecipitation showed a direct interaction of FE65 and Teashirt3 with the promoter region of *CASP4*. Expression studies in postmortem samples demonstrated decreasing expression of Teashirt and increasing expression of caspase-4 with progressive cognitive decline. Importantly, there were significant increases in caspase-4 expression associated with even the earliest neuritic plaque changes in AD. We evaluated a case-control cohort and observed evidence for a genetic association between the Teashirt genes *TSHZ1* and *TSHZ3* and AD, with the *TSHZ3* SNP genotype correlating with expression of Teashirt3. The results were consistent with a model in which reduced expression of Teashirt3, mediated by genetic or other causes, increases caspase-4 expression, leading to progression of AD. Thus the cell biological, gene expression and genetic data support a role for Teashirt/caspase-4 in AD biology. As caspase-4 shows evidence of being a primate-specific gene, current models of AD and other neurodegenerative conditions may be incomplete because of the absence of this gene in the murine genome.

## Introduction

Aβ, the major component of the amyloid fibrils found in AD patients, is a proteolytic product of APP [1]. APP, which is mutated in some early-onset AD patients [2] and overexpressed in others [3], is a type I transmembrane protein and a member of a family of APP-like proteins, also including APLP1 and APLP2. These proteins are proteolytically processed in the extracellular and intracellular domains, including by the γ-secretase protease complex, which leads to the generation of a cytoplasmic fragment (APP intracellular domain/AICD) that has been implicating in intracellular and nuclear signaling [4], [5].

FE65 is an adapter protein with a single WW domain and two phosphotyrosine binding (PTB) domains [6], which binds the YENPTY sequence in the carboxy-terminus of APP through the second PTB (PTB2) of FE65 [7], [8] and which can modulate APP trafficking, and/or enhance the proteolytic processing of APP [9]. FE65 is required for nuclear signaling following the formation of the AICD fragment [5].

It has been shown that the WW domain of FE65 is necessary and sufficient for FE65-dependent transcriptional activation of heterologous reporter genes [6], [10], [11] and that the interaction of APP and FE65 (via the PTB2 domain) at the peripheral membrane is crucial for the full assembly of a transcriptionally active complex [10], [12]. It is therefore of interest to define the role of PTB1 in nuclear signaling, as the proteins that bind to PTB1 might be crucial determinants of the native transcriptional activity of FE65. In this study, we screened for PTB1 binding proteins that might be involved in nuclear signaling and identified the transcriptional core repressor Teashirt as an interactor. We propose that FE65 can function as the core adapter molecule of multi-subunit transcriptional repressor by recruiting both histone deacetylases (via Teashirt) and SET, a component of inhibitor of histone acetyltransferase (INHAT). This complex can in turn silence expression of the *CASP4* gene. Our genetic and expression studies support a role for Teashirt and caspase-4 in AD.

## Results

### Interaction of FE65 with Teashirt proteins

We used the first phosphotyrosine-binding domain (PTB1) of FE65 to carry out comprehensive yeast two-hybrid screens in both mouse and human brain cDNA libraries (Table 1). Multiple interactors were identified from both screens, including previously identified FE65-interactors such as CP2 [13] and calsyntenin [14]. Given our interest in nuclear signaling, we selected putative nuclear proteins including High mobility group 20A (HMG20A), Zinc finger protein 189 (ZNF189), PHD finger protein 1 (PHF1), Teashirt3, and DEAD box 1 (DDX1), and further screened them by co-immunoprecipitation and GST-pulldown assays. As we found that Teashirt3 was positive in both assays (see below), we focused on this protein in further studies. Note that we had identified an interacting clone coding for Teashirt3 from the mouse library, and two Teashirt3 clones from the human library.

**Table 1:** Clones identified in yeast two hybrid screens

Name	Accession number	No. of clones
**Human Brain cDNA Library**		
peroxisomal biogenesis factor 5	NM_000319	7
Alex 3	NM_016607	3
DEAD (Asp-Glu-Ala-Asp) box polypeptide 1	BC012739	4
tripartite motif-containing 8	BC021925	2
teashirt family zinc finger 3	NM_020856	2
dickkopf homolog 3	AB057591	2
leucine rich repeat (in FLII) interacting protein 2	NM_017724	2
deoxythymidylate kinase	NM_012145	2
zinc finger protein 2 homolog	NM_030613	1
FLJ20080	NM_017657	1
microcephaly, primary autosomal recessive 1	NM_024596	1
2,4-dienoyl CoA reductase 1, mitochondrial	NM_001359	1
transcription factor CP2	NM_005653	1
adaptor-related protein complex 2, alpha 1 subunit	NM_014203	1
zinc finger protein 189	AF025770	1
calsyntenin 1	NM_014944	1
C-type lectin domain family 3, member B	NM_003278	1
PHD finger protein 1	NM_002636	1
ligand of numb-protein X 1	BC034737	1
high-mobility group 20A	AF146222	1
**Mouse Brain cDNA Library**		
golgi associated PDZ and coiled-coil motif containing	AF287893	1
DEAD (Asp-Glu-Ala-Asp) box polypeptide 1	BC008570	2
teashirt zinc finger family member 3	NM_172298	1
testis-specific protein, Y-encoded-like 1	BC011213	1
expressed in non-metastatic cells 1, protein	BC005629	1
Similar to Alex3	XM_133800	1

**Figure 1:**
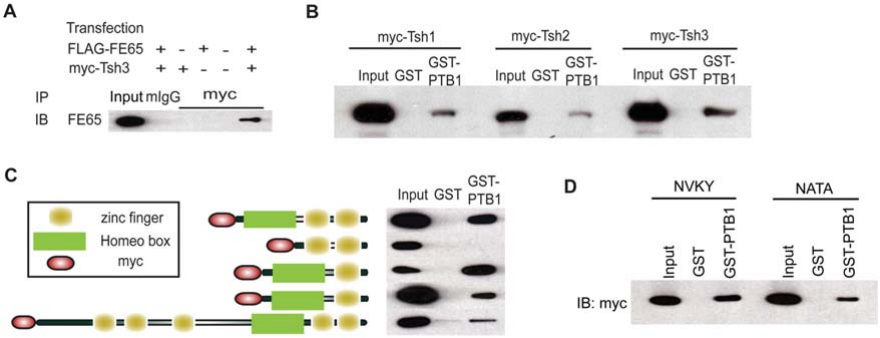
Interaction of FE65 with Teashirt proteins. *A*, Co-immunoprecipitation of FE65 and Teashirt3. H4 neuroglioma cells were transfected with the indicated plasmids (FLAG-FE65 expressing FE65 and/or myc-Tsh3 expressing Teashirt3) and immunoprecipitated (IP) with either mouse IgG (mIgG) or anti-myc antibodies (myc). Immunoprecipitates were analyzed by SDS-PAGE and immunoblotted (IB) with an anti-FE65 antibody. Expression of FE65 in the transfected cells was confirmed by immunoblotting an aliquot of lysate (Input). *B*, GST-pulldown of Teashirt proteins with FE65-PTB1. Lysates from cells expressing the indicated mouse Teashirt constructs (myc-Tsh1, myc-Tsh2, myc-Tsh3 expressing Teashirt1, Teashirt2, and Teashirt3, respectively) were subjected to GST-pulldown assays with either GST alone (GST) or GST-PTB1 and the amounts of recovered protein determined by immunoblotting with an anti-myc antibody. Expression of each construct was confirmed by immunoblotting an aliquot of lysate (Input). *C,* Interaction mapping of FE65 and Teashirt3. Lysates from cells expressing the indicated Teashirt3 constructs were subjected to GST-pulldown assays with either GST alone (GST) or GST-PTB1 and the amounts of recovered protein determined by immunoblotting with an anti-myc antibody. Expression of each construct was confirmed by immunoblotting an aliquot of lysate (Input). *D,* Interaction between GST-PTB1 and myc-tagged wild-type (NVKY) or mutant (NATA) C-terminal constructs of human Teashirt3. Wild-type and mutant constructs were expressed in H4 cells and were subjected to precipitation with either GST alone or with GST-PTB1. After extensive washing, immunoblot analysis using anti-myc antibody (9E10) was performed to test for the presence of the indicated Teashirt3 constructs.

To confirm the FE65-Teashirt3 interaction in mammalian cells, we carried out co-immunoprecipitation of myc-tagged Teashirt3 and FE65 from transiently transfected human neuroglioma (H4) cells (Fig. 1A). Anti-myc antibody, but not control mouse IgG, immunoprecipitated FE65 from lysates expressing both proteins, as detected by anti-FE65 antibody. The COOH-terminal 200 amino acid region of Teashirt3 was identified in the yeast two-hybrid screens. This fragment contains the homeo-box region at the amino-terminus, followed by two zinc finger motifs (see Fig. 1C). In contrast to *Drosophila,* which carries only a single gene encoding Teashirt, it has been reported that the mouse genome contains three structurally related family members, identified as Teashirt1, 2, and 3 [15], [16], all of which include a homeo-box region. We therefore tested if Teashirt1 and Teashirt2 are also able to bind the PTB1 domain of FE65, making use of a GST-pulldown with myc-tagged Teashirt proteins expressed in H4 cells (Fig. 1B). All Teashirt proteins interacted with GST-PTB1 but not GST alone, indicating that FE65 can interact with all Teashirt proteins through PTB1.

To determine which region is responsible for interacting with FE65, deletion constructs of Teashirt3 were prepared and GST-pulldown assays were performed with the deletion constructs and with full-length Teashirt3. Only a construct lacking the homeo-box region failed to interact with GST-PTB1, demonstrating that the interaction with PTB1 domain of FE65 takes place within the homeo-box region (Fig. 1C). For certain PTB interactions, the PTB recognize a consensus NPXY motif, while for other interaction, there is no NPXY motif [17], [18]. Teashirt3 does not contain a canonical NPXY motif within the homeo-box region but there is NVKY sequence. Mutating this sequence to NATA failed to abolish the interaction with FE65 (Fig. 1D) indicating that the interaction between Teashirt3 and FE65 is non-canonical. Other PTB1 domain interactors of FE65 that have been reported to date, such as Tip60, CP2, and calsyntenin, do not contain any canonical motif. Finally, to determine the degree to which FE65 and Teashirt3 colocalize in mammalian cells, we expressed FE65 and/or Teashirt3 in H4 cells and observed that FE65 and myc-tagged Teashirt3 strongly co-localized in the nuclei of H4 cells (data not shown).

### Interaction of endogenous FE65 and Teashirt proteins in native systems

**Figure 2:**
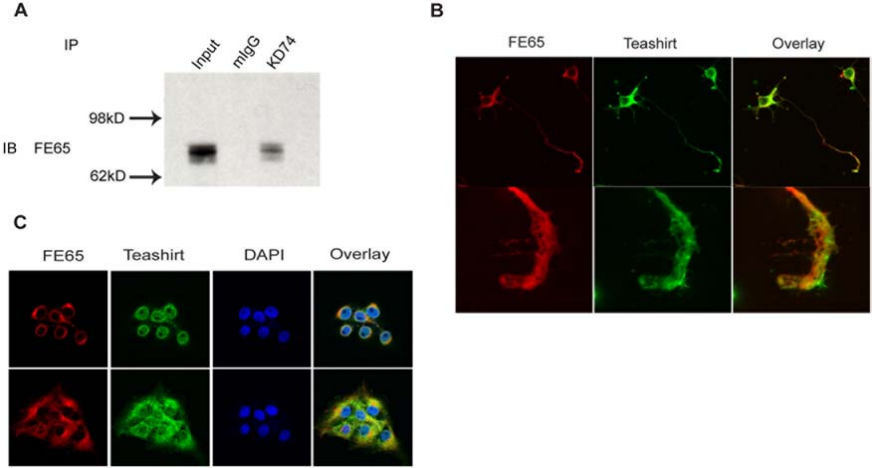
Association of endogenous FE65 and Teashirt proteins. *A*, Co-precipitation of endogenous Teashirt and FE65 from rat cortical neurons. 100 µg of extract derived from rat cortical neurons were subject to immunoprecipition (IP) with either mouse IgG (mIgG) or the KD74 anti-Teashirt antibody and immunoprecipitates were subsequently immunoblotted (IB) with rabbit anti-FE65 antibody. Expression of FE65 in the extract was confirmed by immunoblotting an aliquot of extract (Input). *B*, Colocalization of endogenous Teashirt proteins with FE65 in cell bodies and growth cones from primary neuronal cultures. Rat cortical neurons (3.5 div) were stained with KD74 anti-Teashirt and anti-FE65 antibodies. *C*, Colocalization of endogenous Teashirt proteins with FE65 in H4 cells. H4 cells were immunostained with a rabbit anti-FE65 antibody and a mouse anti-Teashirt monoclonal antibody. DAPI staining was used to highlight nuclei. The upper panels show an optical section in the middle of nuclei while the lower panels show an optical section nearer to the coverslip, where there is greater cytoplasmic extension.

As the validation studies supported a potential physiological interaction between Teashirt3 and FE65, we next developed a series of mouse monoclonal antibodies against Teashirt (identified by their clone identifiers as KD74, VR46 and WR1) and used them to confirm an interaction of endogenous Teashirt proteins and FE65. We first demonstrated that FE65 and Teashirt co-immunoprecipitate from primary neuronal cultures from E18 rat cortices (Fig. 2A). The co-precipitation of these proteins indicates that they are likely to be physically associated in intact cells. We next carried out immunocytochemistry in primary neuronal cultures. When rat cortical neurons maintained for 3.5 div were stained with a Teashirt monoclonal antibody, Teashirt was localized to the cell body as well as to the tips of extending processes, especially in axonal growth cones. When these neuronal cultures were co-immunostained for Teashirt and FE65, both proteins co-localized in growth cones as well as cell bodies, indicating that a binary complex can be formed within these compartments (Fig. 2B). The three monoclonal antibodies showed similar staining of neuronal cultures and we saw a loss of staining in the presence of immunogen, indicating that the staining we observed arose from Teashirt, and not from other proteins or non-specific interactions (data not shown).

We confirmed that our monoclonal antibodies recognize all members of mouse and human Teashirt1–3. Semi-quantitative PCR analysis indicated that rat cortical neuron expresses predominantly Teashirt1 with slightly less expression of Teashirt2 and 3. To explore association of additional endogenous Teashirt proteins with FE65, we studied endogenous FE65 and Teashirt in H4 neuroglioma cells (Fig. 2C). Semi-quantitative PCR analysis indicates that H4 cells predominantly express Teashirt2, with slightly less expression of Teashirt1, while mRNA for Teashirt3 was not detectable (data not shown).

### Teashirt-mediated inhibition of AICD/FE65 transactivation

**Figure 3:**
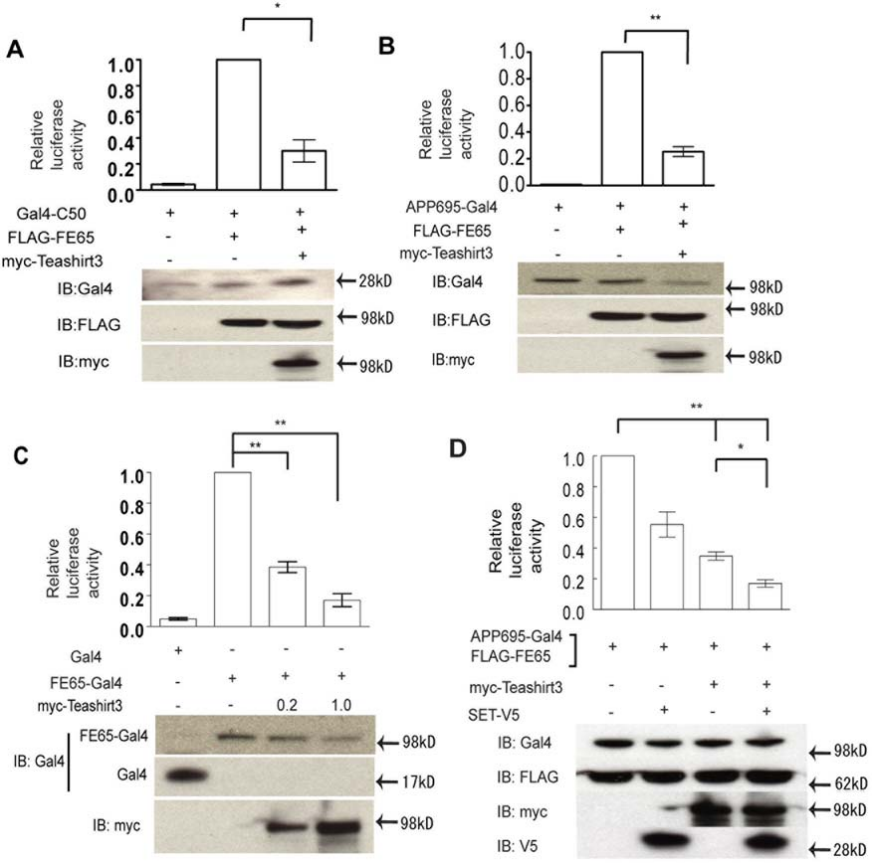
Teashirt3-mediated repression of APP/FE65-activated transcription. *A, B*. Reporter assays for the effects of Teashirt3 on APP/FE65-dependent transactivation. Cells were transfected with Gal4-C50 (A) or APP695-Gal4 (B), in the presence or absence of FE65 and/or Teashirt3, together with luciferase reporter constructs, and the levels of luciferase activity measured. Data are normalized to FE65 and Gal4-C50 (A) or FE65 and APP695-Gal4 (B). *C*, Reporter assay for the effects of Teashirt3 on FE65-dependent transactivation. Cells were transfected with Gal4 alone or with FE65-Gal4 (FE65-Gal4) in the absence or presence of the indicated amounts of Teashirt3 cDNA, together with luciferase reporter constructs, and the levels of luciferase activity measured. Data are normalized to FE65-Gal4 alone. *D*, Reporter assay for the effects of Teashirt3 and SET on APP/FE65-dependent transactivation. Cells were transfected with APP695-Gal4 and FE65, in the presence or absence of SET and/or Teashirt3, together with luciferase reporter constructs, and the levels of luciferase activity measured. Data are normalized to results observed in the presence of FE65 and APP695-Gal4 alone. For A–D, data represent means±SEM of three repeated experiments (with expression levels of transfected proteins for individual experiments shown below each panel) and statistical analyses were performed using two-way ANOVA (P<0.0005 for A–D) followed by Dunnett's T3 post-hoc testing. *, P<0.05; **, P<0.01.

We next carried out reporter analyses with either full length APP or with an AICD construct incorporating the 50 cytoplasmic amino acids of APP produced after ε-cleavage (C50). When FE65 was introduced with either APP-GAL4 or C50-GAL4, there was a 25–150-fold increase in transcriptional activity, but the further introduction of Teashirt3 into the system resulted in a profound down-regulation of the AICD/FE65-mediated up-regulation (Fig. 3A, B). There was no detectable effect of Teashirt3 alone in this system, in the absence of FE65. Immunoblot analysis of lysates showed that expression levels of transfected proteins were not significantly different under different conditions, when averaged over all experiments, indicating that the effect of repression was not due to the difference of expression (representative immunoblots are included in Fig. 3).

Recent studies have suggested that neither γ-secretase cleavage nor nuclear translocation of AICD may be necessary for the transduction of FE65-dependent transactivation in certain systems [10], [12]. To test if Teashirt3 also functions in the absence of AICD or in the absence of an interaction between FE65 and APP, we constructed a fusion protein consisting of Gal4 DNA binding domain fused to FE65. When this reporter system was used, Teashirt3 also inhibited the transactivation by Gal4-FE65 (Fig. 3C).

It has recently been shown that the WW domain of FE65 can interact with SET, one of the main components of inhibitor of histone acetyltransferase (INHAT) [11], [19]. After confirming that FE65 co-precipitated with SET from H4 cell lysates (data not shown), we examined the effects of co-transfecting APP-Gal4 and FE65, with or without Teashirt3 and/or SET, on transcription (Fig. 3D). The addition of either SET or Teashirt3 led to a significant reduction in transactivation. Moreover, the co-expression of both SET and Teashirt3 led to further, significant reduction of transactivation. The effect of repression was not due to the difference of expression, since immunoblot analysis of lysates showed that expression levels of transfected were comparable in different conditions. These data indicated that FE65 can form a transcriptional repressor complex consisting of SET and Teashirt proteins.

### Specificity of Teashirt3 inhibition of transactivation

**Figure 4:**
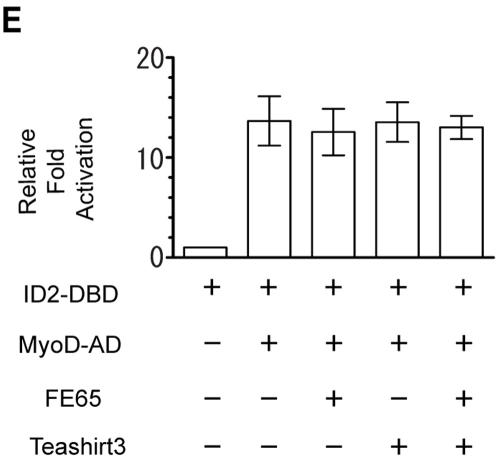
Lack of effect of Teashirt3 on transactivation mediated by ID2-MyoD interation. Dual luciferase assays using ID2-Gal4 DNA binding domain (ID2-DBD) and myoD-Gal4 activation domain (MyoD-AD) were carried out to confirm specificity of Teashirt3 repression to AICD/FE65-mediated transactivation. H4 cells were transfected with the indicated constructs and after assay, luciferase activity was normalized to ID2-DBD alone and shown as relative fold activation. The data shows an average of three independent experiments and statistical analysis using ANOVA show no difference (P>0.1) between any conditions except ID2-DBD alone.

To evaluate whether the effect of Teashirt3 was a general effect on transcription or a specific effect on FE65-mediated gene expression, we made use of the irrelevant interactors ID2 and MyoD, and we observed no effect of Teashirt3, with or without FE65, on transactivation (Fig. 4). This indicated that the effects of Teashirt3 were specific.

### A role for histone deacetylation in Teashirt-mediated transcriptional repression

**Figure 5:**
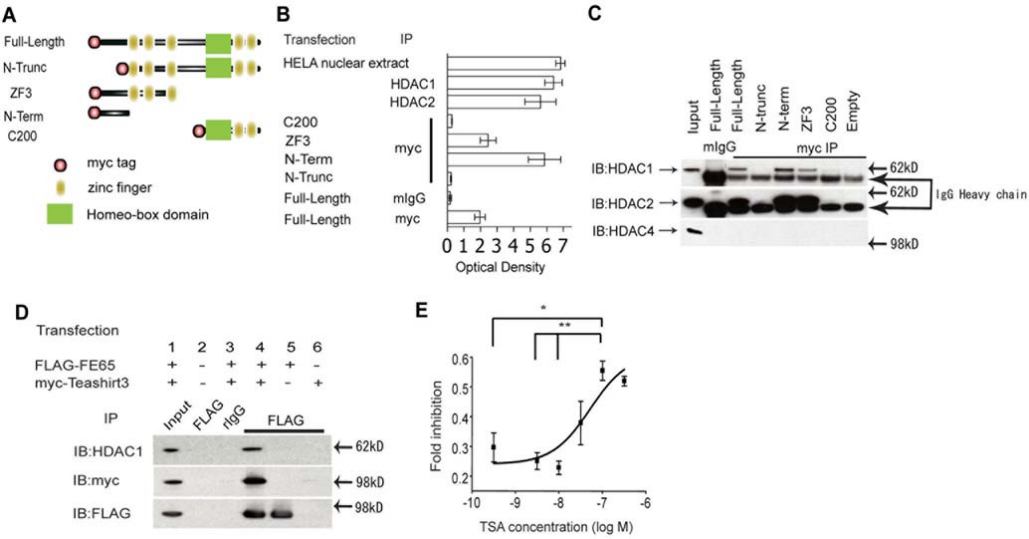
Recruitment of histone deacetylases 1 and 2 by Teashirt3. *A*, Schematic representation of the Teashirt3 constructs used in B and C are depicted. *B,* HDAC assay of Teashirt3 immunoprecipitates. HDAC assays were carried out in nuclear extracts from HeLa cells (HELA) as a positive control (provided by the manufacturer, Biovision) or from H4 cells transfected with the indicated Teashirt3 (Tsh3) constructs (the 5 constructs are shown schematically in C). In addition, HDAC1 and HDAC2 were immunoprecipitated from control cells as additional positive controls. Teashirt3 constructs were precipitated with anti-myc antibodies, or, in the case of full-length Teashirt3, with anti-myc antibodies (myc) or control mouse IgG (mIgG). *C*, HDAC immunoblotting of Teashirt3 immunoprecipitates. The same immunoprecipitates as in B were immunoblotted with the indicated anti-HDAC antibodies. Arrows indicate the HDACs, while HC indicates heavy chain of mouse IgG. Expression of a given HDAC in extracts was confirmed by immunoblotting an aliquot of extract (Input). *D*, Co-immunoprecipitation of Teashirt3, FE65 and HDAC1. H4 cells were nucleofected with FLAG-FE65 and/or myc-Teashirt3 constructs as indicated and immunoprecipitated (IP) with rabbit anti-FLAG antibody (or with rabbit IgG, rIgG). Immunoprecipitates were immunoblotted (IB) with anti-HDAC1, anti-myc, or anti-FLAG M2 antibodies. Expression of exogenous proteins in extracts was confirmed by immunoblotting an aliquot of extract (Input). *E*, Effect of Trichostatin A on Teashirt3-mediated inhibition of transactivation. A reporter assay using Gal4-C50 and FE65 was performed in the presence of increasing concentration of Trichostatin A. Fold inhibition was determined by taking the ratio of the fold activation by FE65 in the presence of Teashirt3 over the fold activation in the absence of Teashirt at each concentration of Trichostatin A. Data represent means±SEM of three repeated experiments. Statistical analyses were performed using two-way ANOVA (P = 0.001) followed by Bonferroni post-hoc testing. *, P<0.05; **, P<0.01.

As transcriptional repression often involves the recruitment of histone deacetylases (HDACs), we first tested if human Teashirt3 can be associated with HDAC activity *in vitro*. When anti-myc antibody immunoprecipitates from H4 cells transfected with Teashirt3 constructs were analyzed for HDAC activity, HDAC activity was recovered in the precipitate (Fig. 5B) (see Fig. 5A for a schematic representation of the constructs used). Constructs containing the amino-terminus of Teashirt3 (i.e., full-length Teashirt3, and the N-term and ZF3 fragments) showed significantly higher HDAC activity, whereas those without the amino-terminus showed background level of activity. [The differences observed in this assay in the level of HDAC activities associated with full-length, N-term, and ZF3 constructs were likely due to the difference in the level of expression in H4 cells, as immunoblot analysis of these constructs with anti-myc antibody showed that the associated HDAC activities of constructs correlated well with the difference in expression among the constructs (data not shown).] These data show that human Teashirt3 can associate with HDAC activity through its amino-terminus *in vitro,* providing a mechanism whereby Teashirt3 is able to function as a transcriptional repressor.

We next sought to identify the HDACs that associated with the amino-terminus of human Teashirt3. In mammals, there are 18 HDACs identified to date and they are assigned into three classes based on the structural similarity to yeast homologues [20], [21]. Class I HDACs are related to yeast Rpd3 and consist of HDAC1, 2, 3, and 8, while class II HDACs are related to yeast Hda and consist of HDAC4, 5, 6, 7, 9, and 10. Class III HDACs require NAD+ as a cofactor and include Sirt1, 2, 3, 4, 5, 6, and 7. We first analyzed the same immunoprecipitates used in the HDAC assay by immunoblot with available anti-HDAC1, HDAC2, HDAC3 or HDAC4 antibodies (Fig. 5C). Full-length human Teashirt3 was immunoprecipitated with endogenous HDAC1 and 2 but not HDAC4, and we found that HDAC3 was not expressed in H4 cells. Consistent with the HDAC activity data, those constructs that precipitated together with endogenous HDAC1 and 2 all contained the amino-terminal domain of Teashirt3, indicating that this region mediates the interaction between Teashirt3 and HDAC. To exclude the possibility that additional proteins were necessary for the interaction between Teashirt3 and HDAC1, recombinant HDAC1 and the amino-terminus of Teashirt3 were expressed and purified from *E. coli* and a direct interaction was confirmed by co-precipitation (data not shown).

To see if FE65 and Teashirt3 form a trimeric complex with endogenous HDAC1, myc-Teashirt3 and FLAG-FE65 were co-nucleofected into H4 cells. FLAG-FE65 was immunoprecipitated from cell lysates using rabbit antibodies to the FLAG epitope and the precipitates were immunoblotted with anti-myc, anti-HDAC1, and anti-FLAG antibodies (Fig. 5D). Endogenous HDAC1 was co-immunoprecipitated with FE65 when both FE65 and Teashirt3 were co-transfected (lane 4), but not when Teashirt3 or FE65 was omitted (lane 5 and 6), confirming that the association of HDAC1 with FE65 is dependent on, and mediated, by Teashirt3.

If the binding of Teashirt3 to FE65 recruits HDAC activity to the complex and mediates transcriptional repression, it would be expected that inhibition of HDAC activity would lead to an inhibition of the Teashirt3-mediated repression of transcription. To test if the transcriptional repression by Teashirt3 in reporter assays is in fact mediated by the action of HDACs, the reporter assay using AICD-GAL4 and FE65 was performed with increasing concentration of the HDAC inhibitor, Trichostatin A (TSA; Fig. 5E). The inhibition of luciferase expression observed in the presence of Teashirt3 decreased with increasing concentration of the inhibitor, confirming that the repressing action of Teashirt3 in the AICD-GAL4 reporter assay was mediated, at least in part, by recruiting Class I and Class II HDACs. As FE65 can simultaneously bind SET and, through Teashirt3, HDACs, this would establish a strongly transcriptionally repressed state of nucleosomes by deacetylating histone amino-terminal tails (through HDAC) and preventing them from being reacetylated (through SET inhibition of histone acetyltransferase).

### The CASP4 gene as a target of the FE65/Teashirt complex

**Figure 6:**
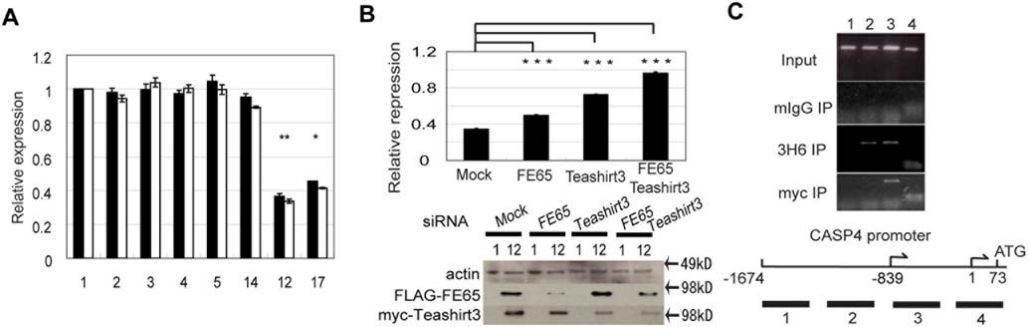
Regulation of caspase-4 expression in H4 cells by Teashirt3 and FE65. *A,* Expression of caspase-4 in stable cell lines. Extracts from indicated cell lines were subjected to qPCR followed by normalization of caspase-4 expression to that of control genes using qBase (version 1.3.5). Three independent experiments were then each normalized to the expression levels of clone 1 (far left) and means and SEM determined. Two distinct probes [identified here as caspase4 (black bars) and caspase4 (white bars)] were used for caspase-4 gene expression. Significant differences in expression were observed for both probes when comparing clones expressing both Teashirt3 and FE65 (clones 12 and 17), as compared to GFP-expressing clones (clones 1–5 and 14). Statistical analyses were performed using two-way ANOVA (P<0.0005) followed by Dunnett's T3 post-hoc testing. (*, P<0.05; **, P<0.01). *B,* Effects of siRNA on caspase-4 expression in stable cell lines. H4 cells stably expressing either GFP (clone 1) or FE65 and Teashirt3 (clone 12) were transfected with siRNA against indicated exogenous proteins. Normalized levels of caspase-4 were quantified as in A, and the fold inhibition of caspase-4 (expressed as the ratio of normalized expression in clone 12 to that in clone 1) in each condition determined. Immunoblot analysis is shown out for a representative experiment. Statistical analyses were performed using ANOVA (P<0.001) followed by Bonferroni post-hoc testing. ***, P<0.001. *C,* Chromatin immunoprecipitation (ChIP) assay with FE65 and Teashirt3. Sonicated chromatin from clone 12 was used for ChIP was subjected to immunoprecipitation with either control mouse IgG, 3H6 anti-FE65, or anti-myc antibodies, and the washed precipitates (as well as an aliquot of the original lysate, indicated as Input) were used for PCR. The scheme below indicates the location of amplified regions in relation to the major transcription start site (1), indicated by an arrow and initiation codon (73) of the *CASP4* gene. Another arrow at −839 position indicates an alternative transcriptional start site.

To identify target genes regulated by the FE65/Teashirt3 complex, lines of H4 cells stably expressing GFP or both FE65 and Teashirt3 were established and analyzed using the Oligo GEArray Human Alzheimer's Disease Microarray, looking for genes that showed reduced expression when both FE65 and Teashirt3 were expressed. Expression from one gene, *CASP4*, which codes for the inflammatory caspase-4, was found to be significantly down-regulated in cells expressing FE65 and Teashirt3, compared to the control cell lines. TaqMan-based quantitative PCR, using two probes directed at caspase-4 as well as probes for control genes, was performed to confirm the GEArray results, demonstrating that the double transfected clones (lines 12 and 17) showed prominent reduction of caspase-4 expression (Fig. 6A).

To further confirm that the reduction of caspase-4 mRNA was mediated by overexpression of FE65 and Teashirt3, both clone 1, expressing GFP, and the double transfected clone 12 were transiently transfected with siRNA against the exogenous FE65 and/or Teashirt3 (Fig. 6B). Relative caspase-4 expression (expressed as the ratio of caspase-4 expression in clone 12 relative to expression in clone 1) was significantly increased when siRNA for either FE65 or Teashirt3 were introduced into clone 12, and the repression was almost completely abolished when both siRNAs were simultaneously introduced, confirming that the reduced expression of caspase-4 observed upon overexpression of FE65 and Teashirt3 was mediated by these proteins. Knock-down of each siRNA targets was confirmed by immunoblot of the cell lysates prepared from the same transfections.

To confirm that there is an association between FE65/Teashirt3 and the *CASP4* gene promoter, chromatin immunoprecipitation (ChIP) assays were performed in clone 12 of the doubly transfected H4 cells (Fig. 6C). Immunoprecipitates with anti-FE65 (3H6), anti-myc, or control mouse IgG were analyzed with four sets of primers designed to amplify the promoter region surrounding *CASP4* transcriptional start site(s) (see scheme). In both the anti-FE65 and anti-myc immunoprecipitates, amplification of a region proximal to the transcriptional start site was observed, indicating that both FE65 and Teashirt3 associated with chromatin near the start site. In the anti-FE65 immunoprecipitates, amplification of a region just upstream of the primary start site was also observed, indicating that conformation of the DNA-protein complex was such that FE65 was also closely associated with this region.

### Altered expression of Teashirt and caspase-4 mRNAs in AD

As we were interested in exploring the relationship of FE65-binding proteins with AD, we analyzed expression of Teashirt and caspase-4, as well as FE65, mRNAs during the course of AD progression. Complementary DNAs were prepared from Brodmann area 28/36 (BA28/36; entorhinal cortex) samples dissected from post-mortem brains of 102 elderly subjects for which there was diagnostic classification based on the Consortium to Establish a Registry for Alzheimer's Disease (CERAD) criteria, as well as information on Clinical Dementia Rating (CDR), neuritic plaque density, and neuropathological measurements (Table 2, 3, 4, 5).

**Table 2:** Demographics of subjects, stratified by diagnosis.

	Control	AD
Number	33	61
Age of Death (years)	79.3±14.3	87.0±8.1
APOE4, Non Carrier/Carrier	27/6	37/24
Tissue pH	6.40±0.22	6.33±0.26
Postmortem interval (min)	498.8±435.5	310.7±213.7
Sex, Male/Female	12/21	43/18

Subjects used in quantitative gene expression study, were grouped by four criteria, including disease status (this Table), CDR score (Table 3), plaque density score (Table 4) and Braak stage (Table 5). In each group we show the number of subjects, age of death (mean±standard deviation), number of APOE4 non-carriers/carriers, sample pH (mean±standard deviation), post-mortem interval (mean±standard deviation), and sex (male/female).

**Table 3:** Demographics of subjects, stratified by CDR.

	CDR 0	CDR 0.5	CDR 1	CDR 2	CDR 3	CDR 4&5
Number	19	12	12	14	19	24
Age of Death (years)	77.2±16.1	85.3±10.6	84.3±10.9	87.9±6.9	86.6±8.6	85.8±9.73
APOE4, Non carrier/Carrier	12/7	9/3	10/2	9/5	14/5	15/9
Tissue pH	6.40±0.21	6.40±0.23	6.24±029	6.37±0.28	6.45±0.22	6.32±0.28
Postmortem interval (min)	594.8±513.3	405.9±330.7	342.8±177.9	340.7±229.9	268.5±161.5	284.5±180.9
Sex, Male/Female	5/14	7/5	5/7	2/12	7/12	6/18

**Table 4:** Demographics of subjects, stratified by plaque density.

	Plaque Density 0	Plaque Density 1	Plaque Density 2	Plaque Density 3
Number	27	17	35	23
Age of Death (years)	76.6±14.1	93.0±5.3	87.5±8.2	82.6±7.9
APOE4, Non Carrier/Carrier	22/5	12/5	24/11	12/11
Tissue pH	6.41±0.24	6.46±0.19	6.32±0.22	6.34±0.34
Postmortem interval (min)	552.9±462.1	236.0±89.7	354.4±252.0	304.0±216.7
Sex, Male/Female	12/15	3/14	7/28	10/13

**Table 5:** Demographics of subjects, stratified by Braak stage.

	Braak 1	Braak 2	Braak 3	Braak 4	Braak 5	Braak 6
Number	8	21	15	8	15	24
Age of Death (years)	80.0±13.1	86.4±10.5	87.5±6.0	88.9±6.9	88.4±8.7	84.5±9.1
APOE4, Non Carrier/Carrier	7/1	18/3	9/6	8/0	10/5	10/10
Tissue pH	6.39±0.29	6.43±0.21	6.35±0.24	6.35±0.16	6.43±0.32	6.30±0.29
Postmortem interval (min)	474.4±329.6	408.1±412.0	351.3±260.7	251.9±92.3	320.5±201.3	302.8±197.4
Sex, Male/Female	3/5	6/15	3/12	1/7	7/8	7/17

**Figure 7:**
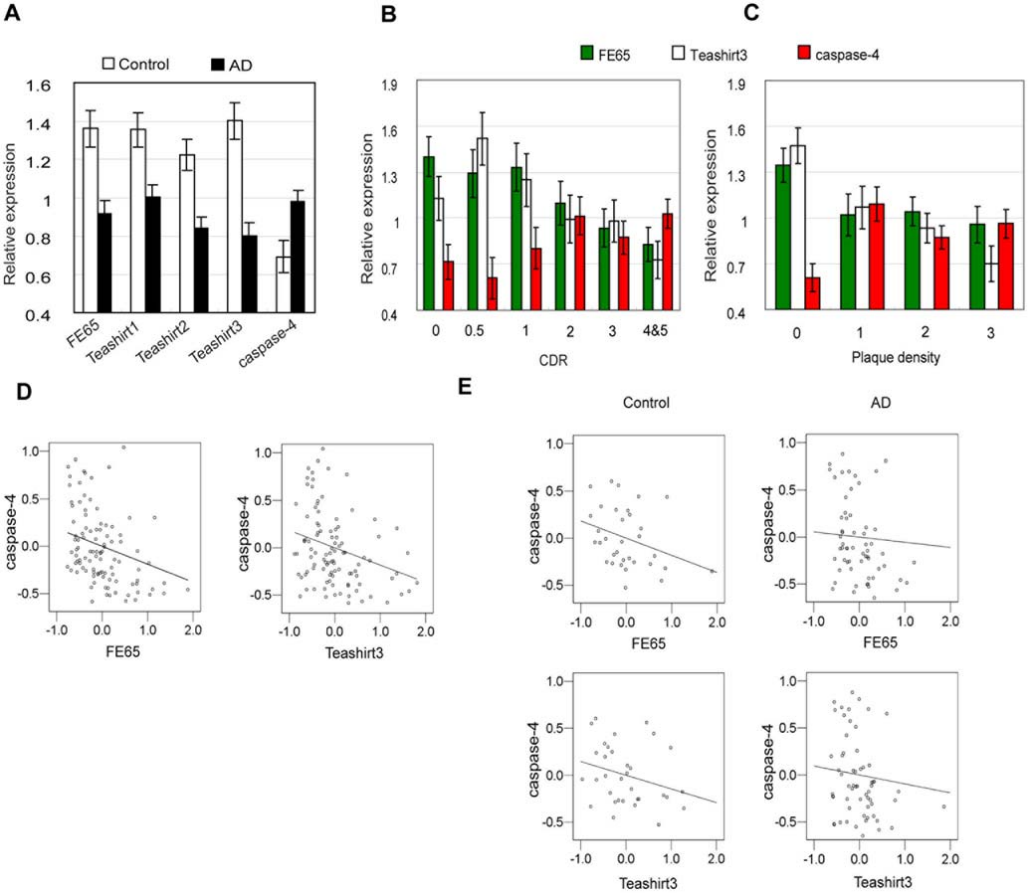
Altered expression of Teashirt and caspase-4 genes in AD. *A,* Altered expression of FE65, caspase-4 and Teashirt gene products in AD. Expression of the indicated genes in BA28/36 was assessed by qPCR, comparing expression in controls with that in subjects with probable or definite AD. All comparisons between cases and controls were significant (P< = 0.003) (see text for a full description of statistical analyses). *B,* Altered expression of FE65, caspase-4 and Teashirt gene products as a function of CDR score. Expression of the indicated genes in BA28/36 was assessed by qPCR, comparing expression in individuals with differing degrees of cognitive impairment as assessed by CDR. For simplicity, results for Teashirt1 and Teashirt2, which paralleled the results for Teashirt3 and are summarized in the text, have been omitted from the figure. *C,* Altered gene expression of FE65, caspases-4 and Teashirt gene products as a function of plaque density. Expression of the indicated genes in BA28/36 was assessed by qPCR, comparing expression in individuals with differing degrees of neocortical neuritic plaque density. For simplicity, results for Teashirt1 and Teashirt2, which paralleled the results for Teashirt3 and are summarized in the text, have been omitted from the figure. *D,* Residuals from linear regressions of caspase-4, FE65, Teashirt1, Teashirt2, and Teashirt3 on APOE4, sex, PMI, and pH were calculated and the associations of residuals between caspase-4 and FE65 or Teashirt genes were plotted, because Pearson correlations of the residuals are equal to partial correlations of the original values. Note that to better visually represent the data, three data points falling outside the graphed area (at positions 1.20, 2.44 and 1.88, −0.46 in left panel and 0.47, 2.44 in right panel), were omitted from the figure but included in the analyses. *E,* To determine whether the significant correlations in C were driven by disease the analyses were re-done in controls (left panels) and in individuals with probable or definite AD (right panels). Note again that to better visually represent the data, data points (at positions 1.89, −0.35 in the upper left panel, 1.30, 2.15 in the upper right panel, as well as 0.74, 2.15 and 1.86, −0.34) in lower right panel, were omitted from the figures but included in the analyses.

Expression of FE65 and all Teashirt family members were significantly reduced in the subjects with definite or probable AD, as compared to control subjects (FE65: F_1, 88_ = 13.490, p<0.0005; Teashirt1: F_1, 88_ = 9.135, p = 0.003; Teashirt2: F_1, 89_ = 13.541, p<0.0005; Teashirt3: F_1, 88_ = 23.399, p<0.0005) (Fig. 7A). In contrast, caspase-4 expression was significantly elevated in subjects with definite or probable AD, as compared to control subjects (F_1, 88_ = 7.081, p = 0.009) (Fig. 7A). As inflammatory processes may change with age, we included age of death as a covariate, and observed that the increase of caspases-4 expression in AD subjects remained significant (F_1, 87_ = 4.375, p = 0.039) [as did the decreases in FE65 (p = 0.002), Teashirt1 (p = 0.013), Teashirt2 (p = 0.001), and Teashirt3 (p<0.0005)].

When gene expression was studied as a function of the degree of cognitive impairment (as measured by CDR), significant difference were seen for FE65 and the Teashirt genes, particularly Teashirt3 (FE65: F_6, 89_ = 3.160, p = 0.007; Teashirt1: F_6, 89_ = 2.613, p = 0.022; Teashirt2: F_6, 89_ = 2.660, p = 0.0020; Teashirt3: F_6, 89_ = 3.262, p = 0.006). Furthermore, when linear regression analyses were performed, significantly negative linear correlations were observed with increasing CDR, indicating reduced expression of these genes with the progression of dementia (FE65: F_1, 94_ = 17.566, p<0.0005, b = −0.128, r = −0.397; Teashirt1: F_1, 94_ = 10.626, p = 0.002, b = −0.094, r = −0.319; Teashirt2: F_1, 94_ = 11.399, p = 0.001, b = −0.092, r = −0.329; Teashirt3: F_1, 94_ = 13.310, p<0.0005, b = −0.124, r = −0.352) (see Fig. 7B). It was notable that p-value/correlation coefficient for FE65 and Teashirt3 were especially robust compared to Teashirt1 and Teashirt2. There was also a significantly positive linear correlation for caspase-4 expression (F_1, 94_ = 6.192, p = 0.015, b = 0.067, r = 0.249) (Fig. 7B), and again this correlation remained significant after controlling for age of death (F_1, 93_ = 5.070, p = 0.027, b = 0.060, r = 0.227).

When gene expression was studied as a function of plaque density (Fig. 7C), significant differences were observed for Teashirt2, Teashirt3 and caspase-4 (FE65: F_3, 98_ = 2.10, p = 0.105; Teashirt1: F_3, 98_ = 2.534, p = 0.062; Teashirt2: F_3, 98_ = 5.952, p = 0.001; Teashirt3: F_3, 98_ = 7.472, p<0.0005; caspase-4: F_3, 98_ = 4.102, p<0.009). It was particularly striking to observe that caspase-4 expression significantly increased when going from a plaque density score of 0, which corresponds to no plaques observed in the 5 cortical regions, to 1, which corresponds to only 1–6 neuritic plaques per mm^2^. After this transition, caspase-4 expression remains elevated as neuritic plaque counts increase to 7–12 (plaque group 2) or >12 (plaque group 3) plaques per mm^2^. Linear regression analysis showed that gene expression of FE65, Teashirt1, Teashirt2, and Teashirt3 were significantly decreased with increasing plaque density (FE65: F_1, 96_ = 4.917, p<0.029, b = −0.117, r = −0.221; Teashirt1: F_1, 96_ = 7.249, p = 0.008, b = −0.128, r = −0.265; Teashirt2: F_1, 96_ = 17.831, p<0.0005, b = −0.398, r = −0.396; Teashirt3: F_1, 96_ = 21.898, p<0.0005, b = −0.444, r = −0.431), whereas that of caspase-4 was significantly increased (F_1, 96_ = 4.011, p = 0.048, b = 0.215, r = 0.200).

When gene expression was studied as a function of the degree of neurofibrillary involvement (as measured by Braak staging), significant difference were seen for FE65 and the Teashirt genes, especially Teashirt2, and Teashirt3 (FE65: F_5, 82_ = 6.328, p<0.0005; Teashirt1: F_5, 82_ = 3.520, p = 0.006; Teashirt2: F_5, 82_ = 5.935, p<0.0005; Teashirt3: F_5, 81_ = 9.841, p<0.0005) (data not shown). There were no significant findings for caspase-4 by ANCOVA (F_5, 81_ = 0.900, p = 0.485). Linear regression analysis showed significantly negative linear correlations, particularly for FE65, Teashirt2, and Teashirt3, with the degree of neurofibrillary involvement (as measured by Braak staging) (FE65: F_1, 86_ = 15.249, p<0.0005, b = −0.123, r = −0.388; Teashirt1: F_1, 86_ = 9.604, p = 0.003, b = −0.096, r = −0.317; Teashirt2: F_1, 86_ = 24.055, p<0.0005, b = −0.133, r = −0.468; Teashirt3: F_1, 85_ = 32.452, p<0.0005, b = −0.176, r = −0.526), indicating reduced expression of these genes with increased neurofibrillary involvement. In contrast, there were no significant findings for caspase-4 (F_1, 86_ = 1.044, p = 0.310, b = 0.114, r = 0.110).

### Relationship between FE65/Teashirt expression and caspase-4 expression

To explore the relationship between FE65 and Teashirt expression with caspase-4 expression stepwise linear regression analyses were performed. Expression of FE65 and Teashirt3 showed significant negative partial correlation with caspase-4 expression (r = −0.229, p = 0.023 and r = −0.239, p = 0.018, respectively) (Fig. 7D), whereas Teashirt1 and Teashirt2 showed non-significant correlation (r = −0.135, p = 0.184, and r = −0.135, p = 0.185, respectively). In order to determine whether the correlations between FE65 or Teashirt3 expression and caspase-4 expression were driven by disease per se, we also carried out exploratory analyses and separately analyzed the correlations for controls and for probable or definite AD (Fig. 7E). In these subgroups, there appeared to be better inverse correlation between caspase-4 and FE65 or Teashirt3 levels in controls than in AD, although the smaller numbers of controls reduced the power to identify significant correlations in that group (correlations between caspase-4 and FE65 or Teashirt3 in the control group were r = −0.326, p = 0.064 and r = −0.278, p = 0.117, whereas in the AD group, these values were r = −0.049, p = 0.707 and r = −0.084, p = 0.522). An inverse correlation in the control samples would indicate that the relationship is not simply a result of disease progression with associated cellular changes. In AD, this relationship breaks down indicating that other factors associated with disease progression (including perhaps cellular changes) have important effects on the expression of caspase-4.

**Figure 8:**
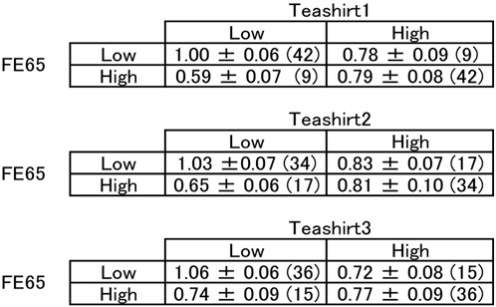
Analysis of the interaction of FE65 and Teashirt expression with caspase-4 expression. FE65 and Teashirt expression in post-mortem samples were dichotomized at the median value and the mean expression of caspase-4 for each of the four groups in the same samples are shown with standard error of mean. Numbers in parenthesis represent the numbers for each group.

A significant interaction between FE65 and Teashirt1 gene expression was observed for the prediction of caspase-4 expression (F_1,94_ = 8.603, p = 0.004). To better understand this interaction, we dichotomized the FE65 and Teashirt1 distributions, and examined the mean expression of caspase-4 in each group (Fig. 8). We noted expression of caspase-4 were high when levels of FE65 and Teashirt1 were low, but in the presence of high levels of Teashirt1, levels of caspase-4 were essentially unchanged with differing levels of FE65. Similar trends were evident when looking at FE65 and either Teashirt2 or Teashirt3 (Fig. 8).

### Association of *TSHZ1* and *TSHZ3* with AD

**Figure 9:**
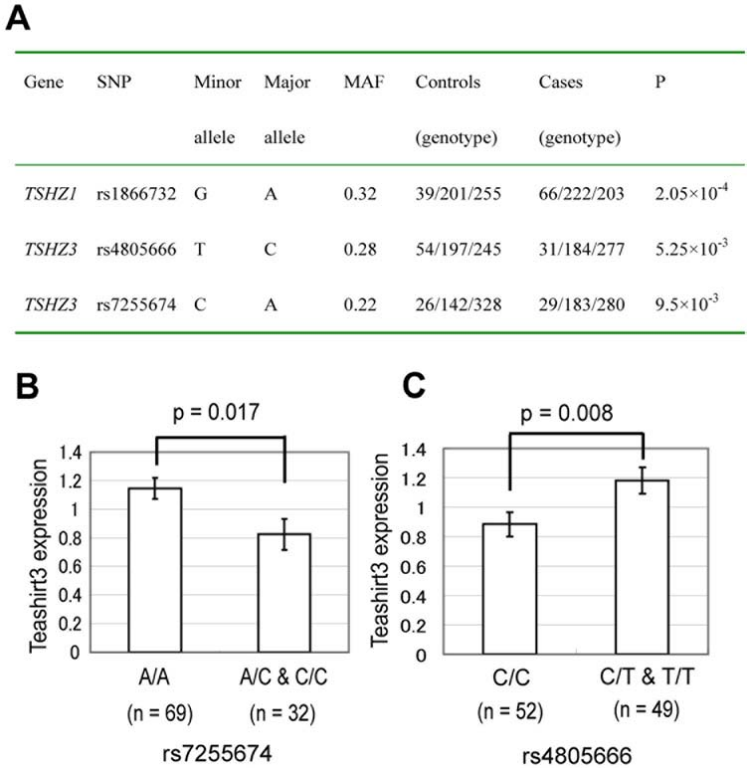
Association of TSHZ genes with AD. *A,* Dense SNPs across the genomic regions harboring the three *TSHZ* genes were evaluated for association. After gene-wide multiple testing correction was carried out using false-discovery rate, with the beta-uniform distribution (FDR-BUM), the three SNPs shown here, with a q-value of <0.20, were identified as significant. *B, C,* Teashirt3 expression in postmortem samples was analyzed as a function of genotype at the TSHZ3 SNPs rs7255674 (B) and rs4805666 (C). Subjects were genotyped and grouped into homozygotes for the major allele and carriers of at least one minor allele. Teashirt3 gene expression was analyzed with ANCOVA, controlling for APOE4, sex, PMI, and pH. Minor allele homozygotes were pooled into heterozygotes due to the limited numbers.

Given the evidence for robust changes in Teashirt expression in AD, and relationship of Teashirt with FE65 signaling, we next explored whether *TSHZ* genes, coding for the Teashirt proteins, might represent susceptibility loci for AD. Dense SNPs across the genomic regions harboring the three *TSHZ* genes were evaluated for association. Several SNPs in each gene showed nominal significance (Supplementary Information S1), using the Armitage test. After gene-wide multiple testing correction was carried out, three SNPs with a q-value of <0.20 were identified as significant (Fig. 9A), including an intronic SNP rs1866732 in *TSHZ1* (P = 2.05×10^−4^), and two SNPs (rs4805666 and rs7255674) in *TSHZ3* (P = 5.25×10^−3^ and 9.5×10^−3^, respectively). The *CASP4* gene was not sufficiently well covered in the genotyping array used and could not be conclusively analyzed.

To determine whether the associated SNPs have an impact on gene expression, we genotyped the three associated SNPs in the 101 postmortem samples for which we had gene expression data. Because of very low numbers of individuals homozygous for the minor allele, we compared expression in individuals homozygous for the major allele with that of individuals with one or two copies of the minor allele. For the two SNPs in *TSHZ3* (rs7255674 and rs4805666), there were significant differences in gene expression between these groups (Fig. 9B, C). It was of great interest to note that for both SNPs, the AD risk allele was associated with decreased Teashirt3 expression, which, based on our cell biological and postmortem studies, we would predict would lead to increased expression of caspase-4.

## Discussion

We have identified Teashirt transcription factors as partners for PTB1 of FE65 and demonstrated that Teashirt3 can repress AICD/FE65 mediated transactivation, consistent with prior evidence that FE65 can function as a transcriptional repressor in certain settings [22]. In *Drosophila*, Teashirt has been shown to be a transcriptional activator of *wing* and *rhomboid* [23], [24], or a transcriptional repressor of homeo-box transcription factor target genes, such as *modifier of variegation* through Scr [25], [26], and wingless target genes, such as *labial* [25] and *ultrabithorax* [27], none of which are represented in the human or mouse genomes. Investigation of the molecular mechanisms of *Drosophila* Teashirt-mediated repression revealed that *Drosophila* Teashirt recruits the transcriptional corepressor CtBP via a PLDLS motif at the amino-terminus [27]. The mouse genome contains three Teashirt genes, Teashirt1, Teashirt2, and Teashirt3, which can rescue *Drosophila tsh* null phenotype, suggesting functional redundancy [28]. Note, however, that the interaction of mouse Teashirt1 with CtBP1 does not account for all the observed transcriptional repression [28], as a significant basal repression activity was observed even after deletion of the CtBP interaction motif, indicating that other factors, aside from CtBP1, contribute to Teashirt-mediated repression in that system. We found that human Teashirt3 associates with HDAC activity and directly interacts with HDACs through its amino-terminus, indicating an additional mechanism for Teashirt-mediated transcriptional repression. It is interesting to note that recent studies have shown that Tip60 can also function as an FE65-dependent repressor [29] and that Tip60 can form complexes with HDAC proteins [30]–[32], indicating that Tip60 also uses similar mechanisms to those we postulate for Teashirt. The relationship between FE65 and Tip60 is interesting in the context of the FE65-Teashirt interaction as both bind PTB1. It is possible that Tip60 and Teashirt compete for FE65 binding, hence regulating transcription. It may be that the choice of partners with which FE65 interacts, either Tip60 or Teashirt, determines the transcriptional role of FE65. In this model, for example, a FE65/Tip60 complex might typically function as a transcriptional activator, while a FE65/Teashirt complex might function as a transcriptional repressor. Our finding that Teashirt proteins localized not only in the nucleus but also in the cytoplasm in neurons, when considered with the presence of nuclear localization signals in all vertebrate members of the Teashirt family (and two nuclear export signals in Teashirt1) raise the possibility that Teashirt proteins distribute between the two compartments in a regulated manner and/or shuttle between them.

We observed that FE65 and Teashirt3 together repress the transcription of *CASP4* gene via a direct interaction as these proteins associate with the *CASP4* gene at the transcriptional start site. In our attempt to obtain further *in vivo* evidence of caspase-4 down-regulation mediated by an FE65/Teashirt complex, we encountered the difficulty that the *CASP4* gene was not represented in the mouse genome (discussed further below), preventing us from utilizing mouse models of AD as well as knockout mice.

The entorhinal cortex (Brodmann area 28/36) is critically involved in memory [33] and subjected to disruption in prodromal stages of AD or mild cognitive impairment [34], [35]. The expression of FE65 and Teashirt family genes were down-regulated in AD, while that of caspase-4 was up-regulated, and we observed negative correlations of gene expression when comparing caspase-4 expression with that of FE65 and Teashirt3. There are many reasons why gene expression would change over the course of AD including loss or gain of specific cell types or functional changes in specific cells. It has been previously shown that at least one other inflammatory caspase (caspase-1), as well as additional caspases, shows increased expression over the course of the disease [36] and much or all of this is likely due to inflammatory processes not directly related to FE65/Teashirt. Of note, however, is that when we examined the correlation of gene expression, comparing caspase-4 expression with that of FE65 and Teashirt, just in the control individuals, we observed evidence for correlations in the controls, and not in individuals with AD, indicating that the cellular change associated with advancing AD are not the exclusive cause for these correlations. It was also interesting to note that caspase-4 expression increased most dramatically with the presence of even just a few neuritic plaques.

A causal role for Teashirt in AD is supported by our genetic association studies. Although genetic association studies in complex disease require replication before they can be accepted, our studies indicate that SNPs in *TSHZ1* and *TSHZ3* show genetic association with AD. Moreover, our studies in postmortem samples are consistent with an effect of *TSHZ3* SNPs on gene expression. More interestingly, we observed that the *TSHZ3* risk alleles are associated with reduced Teashirt3. This suggests a model where reduced expression of Teashirt3 leads to increased expression of caspase-4, which can then contribute to AD risk and pathology. The increase in caspase-4 expression we observed at the earliest stages of neuritic pathology in AD, may contribute to further pathological process in AD progression.

Caspase-4 belongs to a family of inflammatory caspases consisting of caspases-1,-4, -5, -11, and -12 [37]. The mouse genome includes caspase-1, -11, and -12, whereas the human and chimp genomes contain caspase-1, -4, -5, and -12, with caspase-12 typically inactivated in the human genome. Sequence similarity suggests that human caspase-4 and -5 seem to have evolved from gene duplication of a caspase-11-like ancestral gene and there is evidence from studies with LPS that regulation of gene expression of murine caspase-11 parallels that of human caspase-5 [38], [39], with no murine correlate of caspase-4. A primary function of the inflammatory caspase family is the regulation of the inflammatory response. Studies using knock-out mice lacking inflammatory caspases-1, -11, and -12 have shown that these mice were resistant to mouse models of neurodegenerative disease, such as ischemia, amyotrophic lateral sclerosis (ALS), Huntington disease, and Parkinson disease [40]–[44]. This resistance to neuronal cell death is likely due to the lack of inflammatory responses that can have deleterious effects to neurons. Current murine models of AD have surprisingly little neuronal death or synaptic loss, when compared to AD. It is very intriguing to consider whether the absence of a gene whose expression is regulated like caspase-4 in rodents might be partially responsible, something that could be addressed by introducing the human *CASP4* promoter and gene into the murine genome.

**Figure 10:**
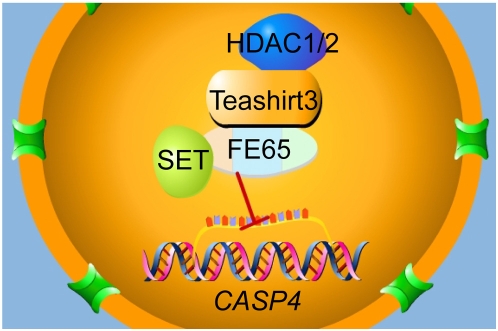
Scheme of proposed complex. We depict the core complex of FE65, SET, and Teashirt3, with the latter binding HDAC1/2, binding to the *CASP4* gene and inhibiting transcription.

As summarized in Figure 10, we have identified Teashirt proteins as FE65-interacting proteins. We have further observed that Teashirt can recruit HDAC, and with FE65 can form a repressor complex that may include SET. Thus, several repressor complexes involving FE65 might form (FE65/Teashirt/HDAC, FE65/SET, or possibly FE65/Teashirt/HDAC/SET), depending on expression levels of these proteins, subcellular compartmentalization, and possibly steric considerations. Our studies indicate that FE65 and/or its associated proteins can possess dual functions in transcriptional regulation: trans-activation by interacting with the histone acetyltransferase Tip60 as previously described [5], [10], [12], and repression by interacting with Tip60 under certain circumstances or by interacting with Teashirt/HDAC and/or SET. The Teashirt family shows altered expression in AD and we have evidence for genetic association for some of these genes with AD. These findings, while intriguing, will benefit from replication in independent cohorts. *CASP4* appears to be one target for the repressor complex centering on FE65. In is interesting to speculate that APP and FE65 may regulate the levels of key inflammatory cascades and to consider that altering the levels or processing of APP might contribute to neuroinflammation and/or neuronal death in neurodegenerative conditions, including AD. As the APP-FE65-Teashirt-caspase-4 pathway not readily studied in current mouse models, its role in neuroinflammation and/or neurodegeneration may be under-recognized.

## Materials and Methods

### Ethical considerations

Rats (purchased from Taconic, NY) were housed and used in compliance with Mount Sinai IACUC. Postmortem brain sample collection was carried out compliance with the IRBs of Pilgrim Psychiatric Center, Mount Sinai School of Medicine, and the Bronx VA Medical Center. In addition, samples for the Collaborative Alzheimer Project (CAP: The Miami Institute for Human Genomics at the University of Miami Medical Center and The Center for Human Genetics Research at Vanderbilt University Medical Center) were collected in compliance with IRBs at each contributing center.

### Cell culture

Human H4 neuroglioma cells were cultured in Dulbecco's Modified Eagle Medium (DMEM) with 10% fetal bovine serum at 37C in 5% CO_2_. Primary cortical neuronal cultures were prepared according to published method [45]. Briefly, cortices of E18 rat embryos were dissected and dissociated by treatment with 0.1% trypsin. Neurons were plated onto coverslips treated with poly-L-lysine in MEM/10% horse serum/0.6% glucose for 2–4 hours until they attached to the coverslips. Coverslips were then placed upside down on astroglial cultures in Neurobasal/B27/2mM glutamine at 37C in 5% CO_2_. Cultures were replenished with fresh Neurobasal medium twice weekly.

### Transfections/nucleofections

Transfections into H4 cells were performed using Fugene6 (Roche, Indianapolis, IN) following the manufacturer's protocol. Cells were seeded for 12 hours at a density recommended by the manufacturer and Fugene6/plasmid DNA mixtures at a ratio of 3∶1 were formed at room temperature for 15–20 minutes and directly added to the medium for 24 hours. H4 cells were nucleofected using an Amaxa Nucleofector (Amaxa Inc, Gaithersburg, MD), following the manufacturer's protocols. Briefly, 5×10^6^ H4 cells were resuspended in 100 µl of supplemented Nucleofector™ solution. Cells were mixed with appropriate plasmid DNA and transferred to a supplied cuvette. All nucleofections were performed using the program U-30 and cells were subsequently transferred to pre-warmed DMEM medium in 6 cm plates.

### siRNA transfections

ON-TARGET*plus SMART*pool siRNAs for rat FE65 (L-091790-01) and human Teashirt3 (L-014119-01) were purchased from Dharmacon. siRNAs were dissolved in RNAse-free 1×PBS at a concentration of 20 µM. H4 cells were plated at 20–30% confluency in 6 cm dishes, and after 12 hours three siRNA transfections were carried out each separated by an additional 12 hours. Each transfection was performed using RNAiMAX (Invitrogen, Carlsbad, CA), following the protocol provided by the manufacturer. Briefly, 5 µl of RNAiMAX and 3 µl of siRNA were added to separate tubes containing 250 µl of Opti-MEM Reduced Serum Medium without serum and then mixed together for 20 minutes. 500 µl of siRNA complex was added to 6 cm plates containing 3ml of DMEM with 10% fetal bovine serum and without penicillin/streptomycin. Media were changed 6 hours after each transfection. Cells were harvested for qPCR analysis 72 hours after the first transfection.

### Construction of plasmids

FLAG-tagged rat FE65 in pCDNA has been previously described [9]. APP695-Gal4 was constructed by overlapping PCR. First, cDNA coding for APP and the DNA binding domain of yeast Gal4 were amplified using primer sets 5′-tatcaagacggaggagatctctg-3′ and 5′-gatagaagacagtagcttgttctgcatctgctcaaa-3′ for APP and 5′-tttgagcagatgcagaacaagctactgtcttctatc-3′ and 5′-gggaaatctagactacgatacagtcaactgtct-3′ for the Gal4 DNA binding domain. A fusion protein of APP and the Gal4 DNA binding domain was amplified using primer set 5′-tatcaagacggaggagatctctg-3′ and 5′-gggaaatctagactacgatacagtcaactgtct-3′ and the fragment was inserted into pCDNA3-APP695 by restriction digestion and ligation. A Gal4-AICD fusion protein was made in the same way using the amino-terminal-directed primer 5′-gggaaaagatctatgctgaagaagaaa-3′ instead of 5′-tatcaagacggaggagatctctg-3′. Full length cDNA encoding human Teashirt3 in the pBluescript II SK(+) vector was obtained from the Kazusa DNA institute (Chiba, Japan). The full-length Teashirt3 insert was amplified using primers 5′-gggaaagaattcggccgaggaggaagcagcag-3′ and 5′-gggaaaagatctctactgcttctctaactc-3′ and cloned into the pCMV-myc vector using EcoRI and BglII sites in frame with an amino-terminal myc epitope. Human HDAC1 cDNA was the generous gift of Dr. S. L. Schreiber (Harvard University, MA), and was amplified using primers 5′-gggaaacatatggcgcagacgcagggcaccc-3′ and 5′-gggaaaaagcttttatttatcatcatcatcttt-3′, and then subcloned into the NdeI and HindIII sites of the pET28a vector, which contains an amino-terminal 6×His tag. Mouse Teashirt constructs were generous gifts of Dr. L. Fasano (CNRS Université de la Méditerranée, France).

### Purification of bacterial recombinant proteins

Chemically competent BL21(DE3)pLysS *E. coli* cells (Stratagene, La Jolla, CA) were transformed with the pGEX2T-hTeashirt3-ZF12 or pET28a-HDAC1 vectors using the manufacturer's protocol, and plated on LB plates with appropriate antibiotics. A single colony was inoculated in 10 ml of LB medium with antibiotics, grown overnight and seeded into 500 ml of LB medium. The bacterial cultures were grown to an optical density of 0.6 and expression of recombinant proteins were induced by the addition of isopropyl-beta-D-thiogalactopyranoside (IPTG) to a final concentration of 0.5 mM for 4 hours at 37C. The glutathione S-transferase (GST) fusion proteins were harvested by centrifuging bacteria at 5,000 g for 15 minutes and the resultant pellets were resuspended in lysis buffer containing 1× phosphate buffered saline (PBS)/1% tritonX-100/Complete protease inhibitor cocktail (Roche, Indianapolis, IN). Cells were lysed in a French press (SIM AMINCO Spectronic Instruments, Rochester, NY) and centrifuged at 10,000g for 15 minutes to precipitate the debris. Supernatants were incubated with Glutathione SepharoseTM 4B (GE Healthcare, Piscataway, NJ) on a rotator for 4 hours at 4C and washed 3 times with wash buffer containing PBS/protease inhibitor cocktail.

For the production of antibodies, antigen GST-hTsh3(184–324) was eluted with a buffer containing 1× PBS/6M guanidine hydrochloride/10mM DTT with incubation at 70C for 4 hours. The purified antigen was dialyzed against 1× PBS and concentrated using Centriprep YM-10 from Millipore (Billerica, MA). For the purification of His-tagged HDAC1, cells were resuspended in lysis buffer containing 50mM sodium phosphate (pH8.0)/300mM NaCl/10mM imidazole and lysed with three rounds in the French press. Crude lysate was cleared by centrifugation at 10,000g for 15 minutes and the supernatant was incubated with 0.5ml of Ni-NTA agarose (Qiagen, Valencia, CA) for 4 hours. The beads were precipitated and washed three time with wash buffer containing 50mM sodium phosphate (pH 8.0)/300mM NaCl/20mM imidazole and eluted with elution buffer containing 50mM sodium phosphate (pH 8.0)/300mM NaCl/250mM imidazole.

### Antibodies

The 172 rabbit polyclonal antibody directed against the WW domain of FE65 has been previously described [9]. The 9E10 mouse monoclonal antibody against the myc epitope tag was obtained from the Mount Sinai Hybridoma Facility. The 2E10 mouse monoclonal antibody against HDAC1 and the 3F3 antibody against HDAC2 were obtained from Upstate (Lake Placid, NY). A rabbit polyclonal antibody against HDAC4 (#2072) was obtained from Cell Signaling Technology (Danvers, MA). Monoclonal Teashirt antibodies were generated by using GST fused to amino acids 184–324 of human Teashirt3 as the antigen. The recombinant protein was expressed in the BL21(DE3)pLysS strain of *E. coli* and purified as described above. Injection of the antigen into mice, generation of hybridomas, clone screening, and clone isolation were performed in the Mount Sinai Hybridoma Shared Research Facility.

Antigen pre-treatments were performed by incubating purified antibodies with the antigen immobilized to glutathione sepharose beads for 24 hours followed by the removal of beads using 0.22 µm spin-X cellulose acetate filters.

### Yeast two-hybrid screening

Two-hybrid screening was carried out together with Dualsystems Biotech (Zurich, Switzerland). The PTB1 domain of rat FE65 (amino acids 360–524) was cloned into the DUALhybrid bait vector in frame with the LexA DNA-binding domain. After validating that the PTB1 domain did not, by itself, activate reporter gene expression, adult mouse and fetal human brain cDNA libraries were screened (Clontech, Mountain View, CA) resulting in 41 and 38 positive clones respectively. Bait dependency of these clones was confirmed by retransforming them into strains carrying either the FE65 PTB1-LexA construct or a control vector.

### GST-pulldown

Purified GST-fusion constructs bound to Glutathione agarose beads were incubated with H4 cell lysate expressing indicated expression constructs. Beads were extensively washed with wash buffer consisting of phosphate buffer saline, pH7.4 and 1% TritonX-100. The beads were then boiled in SDS gel loading buffer and analyzed by SDS-PAGE and immunoblot.

### Coimmunoprecipitation of FE65 and Teashirt

FLAG-FE65 and myc-Teashirt3 (human) were co-transfected into H4 cells on 100mm dish using Fugene6 for 24 hours and harvested in lysis buffer containing 1× PBS/0.5% TritonX-100/protease inhibitor cocktail (Roche). Alternatively, for primary neuronal cultures, cells were lysed in the presence of 1× PBS/2% Triton X-100/protease inhibitor cocktail (Roche).

Lysates were sonicated using a Misonix microson sonicator (Misonix, Inc. Farmingdale, NY) and centrifuged at 16,000 g for 1 hour. 500 µg of lysate was immunoprecipitated using 5 µl of 173 rabbit polyclonal anti-FE65 antibody followed by the addition of 20 µl of protein A agarose (Invitrogen, Carlsbad, CA). The immunoprecipitate was washed 3 times using lysis buffer and eluted by boiling in SDS sample loading buffer containing 1M Tris-HCl (pH6.8)/10% SDS/50% Glycerol/200mM β-mercaptoethanol/bromophenol blue. Samples were run on a 10% NOVEX SDS-PAGE gel (Invitrogen, Carlsbad, CA) and transferred to PVDF membranes. Immunoblotting was carried out using 9E10 anti-myc antibody, followed by secondary anti-mouse HRP antibody and detection using the SuperSignal West Pico Chemiluminescent Substrate (Pierce, Rockford, IL).

### Coimmunoprecipitation of FE65 and SET

V5-tagged SET and FLAG-tagged FE65 were co-transfected into H4 cells and cell lysates were immunoprecipitated with anti-FLAG agarose (or anti-myc-agarose as a negative control). Immunoprecipitates were analyzed by SDS-PAGE and immunoblotted with mouse monoclonal anti-V5 antibody or 173 rabbit polyclonal anti-FE65 antibody.

### Immunocytochemistry

Cortical neuronal cultures were fixed in 4% formaldehyde/0.12M sucrose in PBS pre-warmed to 37C for 10 minutes and subsequently permeabilized in 0.1% TritonX-100 for one minute. Cortical neuron had no detectable endogenous peroxidase activity such that H_2_O_2_ treatment was not necessary. Cultures were incubated with 1% blocking buffer (Invitrogen, Carlsbad, CA) for 1 hour at room temperature, and then with the indicated antibodies (1∶200 for anti-myc, 1∶200 for 173 anti-FE65, and 1∶60 for anti-Tsh antibodies). After three washes with 1× PBS/0.2% BSA, cells were incubated with secondary antibody (1∶100 for TexasRed anti-rabbit IgG, FITC anti-mouse IgG, or horse raddish peroxidase (HRP) anti-mouse/rabbit IgG). For tyramide staining for endogenous Teashirt in H4 neuorglioma and neuron cultures, either AlexaFluor 488 or AlexaFluor594 dyes (Invitrogen, Carlsbad, CA) were diluted in 1∶100 in amplification buffer with 0.015% H_2_O_2_ and incubated for 5–10 minutes. Coverslips were mounted onto slides with vectashield mounting medium (Vector, Burlingame, CA).

H4 neuroglioma cells grown on coverslips in 12 well plate, fixed in 4% formaldehyde in PBS for 15 minutes and permeabilized in 0.5% TritonX-100 for 20 minutes. For staining using tyramide-AlexaFluor dye, endogenous peroxidase activity was eliminated by 1% H_2_O_2_ treatment for 1 hour.

### Confocal microscopy

Fluorescent images were captured using either a Leica TCS-SP (UV) confocal microscope or a Zeiss LSM 510 META in the Mount Sinai Shared Research Facility.

### Reporter assays

H4 neuroglioma cells were seeded on 12 well plates at the density of 10^5^ cells per well, and grown for 12 hours at 37C. Cells were transfected using Fugene6, with 1 µg/well of reporter gene plasmid pFR-Luc and 0.1 µg/well of control plasmid phRL-Null, along with 1 µg/well of each expression plasmid indicated. 24 hours after transfection, cells were lysed in 250 µl of 1× lysis buffer (Promega, Madison, WI) and vigorously shaken on a shaker for 15 minutes. 25 µl each of lysate from a single well was distributed into three wells and firefly and renilla luciferase activities were sequentially measured with L-Max luminometer which injected 100 µl each of LAR II buffer and Stop&Gro buffer (Molecular Devices, Sunnyvale, CA). Transfections were performed in triplicate. For studies with the HDAC inhibitor Trichostatin A, 6 hours after transfection, 1mM Trichostatin A (T8552, Sigma, St. Louis, MO) in dimethylsulfoxide was diluted into the medium to the indicated concentration and cells were treated for another 24 hours. Cells were harvested and luciferase activities were measured as above. Fold inhibition was determined by taking the ratio of the fold activation by FE65 in the presence of Teashirt3 over the fold activation in the absence of Teashirt3 at each concentration of Trichostatin A.

### HDAC assays

Myc-tagged Teashirts3 constructs were transfected into H4 cells in 10 cm dishes for 24 hours and harvested in lysis buffer containing 1× PBS, 0.5% Triton-X 100 and Complete Protease Inhibitor cocktail (Roche). Recombinant proteins were immunoprecipitated using 5 µg of 9E10 anti-myc monoclonal antibody and 20 µl of protein G agarose (Roche) and washed 2 times with lysis buffer. HDAC assays were performed on the immunoprecipitates using the Biovision Colorimetric Assay Kit (K331-100) following the manufacturer's protocol. Briefly, 10 µl of 10× buffer and 5 µl of substrate were added to the lysate to a final volume of 100 µl. The reaction mixture was incubated at 37C and then stopped by adding 5 µl of developing solution.

### Oligo GEArray

For cRNA synthesis, H4 neuroglioma cell lines stably expressing GFP or FLAG-FE65 together with myc-Teashirt3 were harvested and total RNA purified using Qiagen RNA easy kits following the manufacturer's protocol. cRNAs were synthesized and purified using True-Labeling-AMP^™^ 2.0 kit (Superarray, Frederick, MD). Briefly, 2.5 µg of total RNA was mixed with TrueLabeling Primer and heated at 70°C for 10 minutes. RNase Inhibitor, cDNA enzyme mix, and 5× synthesis buffer were then added and the mixture heated at 42°C for 50 minutes, followed by 5 minutes heat inactivation at 75°C. cRNAs were synthesized by adding 2.5× cRNA synthesis buffer, Biotin-16-UTP, and RNA polymerase to the reaction mixture followed by incubation at 37°C overnight. An average of 10 µg of cRNAs were recovered after column purification.

For hybridization, membranes were prewet with deionized water and prehybridized with warm GEAhyb Hybridization Solution at 60°C for 2 hours. 4 µg of biotin labeled cRNAs were mixed in Hybridization Solution and incubated with membranes at 60°C overnight. Membranes were washed first with 5ml of Buffer 1 (2×SSC, 1%SDS) and then washed with 5ml of Buffer 2 (0.1×SSC, 0.5%SDS) at 60°C for exactly 15 minutes. For chemiluminescent detection, GEAblocking Solution Q was added to the membranes with 40 minutes incubation at room temperature. Alkaline phosphatase conjugated with streptavidin diluted in 1× Buffer F was then added to the membrane for 10 minutes, followed by extensive washing with 1× Buffer F four times. The membranes were equilibrated with Buffer G before the chemiluminescence detection reagent was added for 5 minutes. Chemiluminescent signals were detected and captured as 8 bit TIFF files using a Biorad ChemiDoc XRS. For image analysis, acquired array images were loaded to GEArray Expression Analysis Suite software and further analyzed.

### Quantitative PCR analysis in cell lines

Total RNA was purified using Qiagen RNeasy kit and cDNA was synthesized using Superscript®III first-strand synthesis kit (Invitrogen, Carlsbad, CA)**.** Taqman® probes (Applied Biosystems, Foster City, CA) included control probes (β-glucuronidase, Hs99999908_m1 β2-macroglobulin, Hs99999907_m1; and RPLP0, Hs99999902_m1), and two probes for caspase-4 (Hs00233438_m1 and Hs01031951_m1). qPCR were run three times with TaqMan® Universal PCR Master Mix (430437, Roche) with an ABI 7900HT real-time machine at the Mount Sinai Quantitative PCR facility, with each run containing triplicate samples. Acquired data were loaded onto qBase ver.1.3.5 software for data quality control and normalization. Normalized gene expression values of each clone were then additionally normalized to GFP clone1 to simplify analysis across experiments.

### Chromatin immunoprecipitation (ChIP)

H4 neuroglioma cells expressing FLAG-FE65 and myc-Teashirt3 were subjected to cross-linking in 1% formaldehyde for 10 minutes at room temperature, followed by incubation with 125 mM glycine for 5 minutes to stop cross-linking. Cells were washed twice with cold phosphate buffer saline and harvested in hypotonic buffer containing 10 mM sodium citrate, pH 6.5, 10 mM EDTA, and protease inhibitor cocktail (Roche). Cells were centrifuged and resuspended in lysis buffer containing 50 mM Tris, pH 8.1, 10 mM EDTA, 1% SDS, and protease inhibitor cocktail. Sonication was carried out in 15 ml polypropylene tube using Bioruptor (diagenode, Belgium) with 3 repeats of an on/off cycle (30 seconds on/30 seconds off). Sonicated lysates were diluted in 10 volumes of buffer (0.01% SDS, 1.1% Triton X-100, 1.2 mM EDTA, 16.7 mM Tris-HCl, pH 8.1, 167 mM NaCl and protease cocktail) and incubated with protein agarose A/G and BSA (1.5 mg/ml). After centrifugation, supernatants were incubated with indicated antibodies overnight and subsequently incubated with 40 µl of protein agarose A/G. Precipitates were serially washed with the following buffers: (1) Low Salt buffer (0.1% SDS, 1% Triton X-100, 2 mM EDTA, 20 mM Tris-HCl, pH 8.1, 150 mM NaCl); (2) High Salt buffer (0.1% SDS, 1% Triton X-100, 2 mM EDTA, 20 mM Tris-HCl, pH 8.1, 500 mM NaCl); (3) LiCl buffer (0.25 M LiCl, 1% NP40, 1% deoxycholate, 1 mM EDTA, 10 mM Tris-HCl, pH 8.1); and, (4) TE buffer (10 mM Tris-HCl, 1 mM EDTA, pH 8.0)**.** Precipitated chromatin DNA was released by incubating with protease K at 65°C for 6 hours, purified using Qiagen PCR purification kit, and analyzed by PCR. The sequences of the primer sets used to amplify the caspase-4 promoter regions were as follows. For region 1, 5′-aagctctcacgctgtgtttctcag -3′ and 5′-agataaatccacaaagatgacgaa -3′; for region 2, 5′-cttttttgttgatgctgtgttatt -3′ and 5′-aagggatagccagactctttcctt -3′; for region 3, 5′-caggtgctctgtaccagggaaatg -3′ and 5′-gctggattagaatccccactagcc -3′; for region 4, 5′-gagatcaggaagagaagagacaga -3′ and 5′-cagcctctgtccttttttacagcg -3′.

### Quantitative PCR analysis in post-mortem brain samples

Subject selection, cognitive assessment, neuropathological assessment, and stratification were previously described [46]. Neuritic plaque density was quantified in 5 cortical region Brodmann area 9 (BA9; middle frontal gyrus), Brodmann area 45/47 (BA45/47; orbital frontal gyrus), Brodmann area 21/22 (BA21/22; superior temporal gyrus), Brodmann area 39 (BA39; inferior parietal cortex), and Brodmann area 17 (BA17; calcarine cortex), as previously described [47]. Frozen post-mortem brain tissue from the parahippocampal gyrus (Brodmann area 36) of subjects with or without a CERAD Alzheimer's diagnosis [48] (control, N = 33; definite AD, N = 52; probable AD, N = 9; possible AD, N = 8) were obtained from the Mount Sinai/Bronx Veterans Administration (VA) Medical Center/Department of Psychiatry Brain Bank. Normal controls had no history of any psychiatric or neurological disorders and no discernible neuropathological lesions. The Institutional Review Boards of Pilgrim Psychiatric Center, Mount Sinai School of Medicine, and the Bronx VA Medical Center approved all assessment and post-mortem procedures. Total RNA extraction and reverse transcriptase (RT) reactions were performed after DNAse treatment and template RNA quality, including degradation and DNA contamination, was controlled as described previously [49]–[51]. Taqman® probes for endogenous control gene are the same as described above and experimental probes are (Teashirt1, Hs00362334_s1; Teashirt2, Hs00542836_m1; Teashirt3, Hs02379784_s1; FE65, Hs01013014_m1; and caspase-4, Hs00233438_m1). The reactions were carried out as described above. Each gene expression values were calculated using the comparative 2^−ΔΔ^Ct method described previously [52] and additionally normalized to geometric mean of the control genes.

For the quantitative analyses of gene expressions in post-mortem samples (FE65, caspase-4, Teashirt1, Teashirt2, and Teashirt3), analyses of covariance (ANCOVA) were performed to compare gene expression for different diagnoses, clinical dementia rating scale (CDR) levels, plaque density, and Braak stages. The covariates were post-mortem interval (PMI), sex, APOE4 genotype, and sample pH. However, a covariate was omitted from an analysis if there was significant heterogeneity of the groups' regression coefficients. The figures display the adjusted estimates of the mean and standard error of the mean from ANCOVA. For CDR and Braak stage analyses, stepwise regression was used to test linear association of gene expression, controlling for the same covariates as in ANCOVA. Stepwise linear regression was used to assess the partial correlations of FE65, Teashirt1, Teashirt2, and Teashirt3 gene expression with caspase-4 expression, controlling for APOE4 genotype, sex, sample pH, and PMI. A subsequent step evaluated the interactions of FE65 with Teashirt1, Teashirt2, and Teashirt3 gene expression for predicting caspase-4 expression.

### Association analysis

The case-control sample set, genotyping, and quality control have been described previously [53]. Briefly, samples were derived from the Collaborative Alzheimer Project (CAP: The Miami Institute for Human Genomics at the University of Miami Medical Center and The Center for Human Genetics Research at Vanderbilt University Medical Center) and written informed consent was obtained from all participants in agreement with protocols approved by the institutional review board at each contributing center. The final sample included DNA from a total of 988 Caucasian individuals, including 492 AD cases aged 72.9 years +/− 6.6 years (61% female) and 496 cognitive controls aged 74.3 years +/− 6.5 years (63% female). For inclusion, each affected individual met the NINCDS/ADRDA criteria for probable or definite AD and had an age at onset greater than 60 years of age. Cognitive controls were spouses, friends, and other biologically unrelated individuals who were frequency age and gender matched to the cases, and all were from within the same clinical catchment areas. All cognitive controls were examined and none showed signs of dementia by history and upon interview. Additionally, cognitive controls each have a documented Mini-Mental State Exam (MMSE) of at least 27 or Modified Mini-Mental State Exam (3MS) of at least 87. Genotyping was performed using the Illumina Beadstation and Illumina 550K HumanHap Beadchip following recommended conditions, with the exception of requiring the more conservative gencall score of 0.25 and has been described.

### Statistical analyses

SPSS version 12.0 (SPSS, Inc., Chicago, IL, USA) was used for all analyses. Where indicated, ANOVA was carried out, followed by either Bonferroni or, for datasets that included a normalized reference group Dunnett's T3, post-hoc tests (the latter assuming unequal variance). ANCOVA and linear regression was carried out in the analyses of gene expressions in post-mortem samples.

Association analyses using the Armitage trend test [54] and gene-wide multiple testing correction [55]. This method tests for a linear trend in the number of alleles at a single locus. That is, two copies of an allele have more of an effect than one copy, which in turn has more of an effect than no copies. The effect is in the same direction for each genotype. This test is equivalent to the score statistic from a logistic regression model with no covariates. Gene-wide multiple testing correction was applied with a false-discovery rate, using the beta-uniform distribution and SNPs with q-values less than a 0.20 false discovery rate were declared significant.

## Supporting Information

Supplementary Information S1 (0.58 MB DOC)
