# Supplementary material for: FE65 Binds Teashirt, Inhibiting Expression of the Primate-Specific Caspase-4
Source: PLoS One. 2009 Apr 3;4(4):e5071. doi: 10.1371/journal.pone.0005071 (PMC2660419; doi:10.1371/journal.pone.0005071)
Supplement: Supplementary Information S1 — (0.58 MB DOC) [file pone.0005071.s001.doc]

| **TSHZ1**  **PROBE** | **CHROM** | **START_LOCATION** | **Mbp Location** | **ARMIT_TREND** | **p-value** |
| --- | --- | --- | --- | --- | --- |
| RS596996 | 18 | 71004971 | 71.004971 | 0.0751 | 0.7840 |
| RS7231415 | 18 | 71008111 | 71.008111 | 1.2860 | 0.2568 |
| RS6566060 | 18 | 71009457 | 71.009457 | 0.2979 | 0.5852 |
| RS787583 | 18 | 71013458 | 71.013458 | 0.4328 | 0.5106 |
| RS4643425 | 18 | 71017279 | 71.017279 | 0.0212 | 0.8843 |
| RS906508 | 18 | 71029953 | 71.029953 | 0.0018 | 0.9658 |
| RS629126 | 18 | 71034991 | 71.034991 | 0.2812 | 0.5959 |
| RS629081 | 18 | 71035025 | 71.035025 | 0.3105 | 0.5774 |
| RS1132845 | 18 | 71041457 | 71.041457 | 0.1394 | 0.7089 |
| RS7230037 | 18 | 71043268 | 71.043268 | 0.1458 | 0.7026 |
| RS2639989 | 18 | 71044927 | 71.044927 | 0.0400 | 0.8415 |
| RS2581651 | 18 | 71055030 | 71.055030 | 4.9390 | 0.0263 |
| ***RS1866732*** | ***18*** | ***71062175*** | ***71.062175*** | ***13.7806*** | ***0.0002*** |
| RS2639966 | 18 | 71067174 | 71.067174 | 0.6347 | 0.4256 |
| RS17056685 | 18 | 71071184 | 71.071184 | 0.2138 | 0.6438 |
| RS6566062 | 18 | 71072851 | 71.072851 | 0.1274 | 0.7212 |
| RS8086757 | 18 | 71074069 | 71.074069 | 0.2636 | 0.6076 |
| RS17056699 | 18 | 71074256 | 71.074256 | 0.1678 | 0.6821 |
| RS17056701 | 18 | 71074702 | 71.074702 | 2.0231 | 0.1549 |
| RS12955812 | 18 | 71075286 | 71.075286 | 0.4154 | 0.5192 |
| RS9959886 | 18 | 71077061 | 71.077061 | 0.2001 | 0.6547 |
| RS17056720 | 18 | 71078018 | 71.078018 | 0.0058 | 0.9392 |
| RS11875615 | 18 | 71091216 | 71.091216 | 0.1734 | 0.6771 |
| RS4891252 | 18 | 71103295 | 71.103295 | 0.1203 | 0.7287 |
| RS1978899 | 18 | 71103677 | 71.103677 | 1.5455 | 0.2138 |
| RS6566067 | 18 | 71104943 | 71.104943 | 0.1203 | 0.7287 |
| RS4891254 | 18 | 71112948 | 71.112948 | 0.4881 | 0.4848 |
| RS8085298 | 18 | 71113925 | 71.113925 | 1.3241 | 0.2499 |
| RS2581644 | 18 | 71116477 | 71.116477 | 2.4746 | 0.1157 |
| RS2581643 | 18 | 71116512 | 71.116512 | 0.5496 | 0.4585 |
| RS2639972 | 18 | 71117467 | 71.117467 | 0.1017 | 0.7497 |
| RS2581642 | 18 | 71117893 | 71.117893 | 0.3144 | 0.5750 |
| RS2581641 | 18 | 71119261 | 71.119261 | 0.0832 | 0.7730 |
| RS3826609 | 18 | 71126665 | 71.126665 | 0.7396 | 0.3898 |
| RS3809997 | 18 | 71127874 | 71.127874 | 4.7280 | 0.0297 |
| RS9953004 | 18 | 71131735 | 71.131735 | 1.9288 | 0.1649 |
| RS4383238 | 18 | 71132026 | 71.132026 | 0.8732 | 0.3501 |
| RS2581639 | 18 | 71132478 | 71.132478 | 0.0722 | 0.7882 |
| RS2639982 | 18 | 71134403 | 71.134403 | 1.9596 | 0.1616 |
| RS10871570 | 18 | 71135956 | 71.135956 | 0.0208 | 0.8853 |
| RS9946654 | 18 | 71140324 | 71.140324 | 0.3498 | 0.5543 |
| RS12962160 | 18 | 71142169 | 71.142169 | 0.3498 | 0.5543 |
| RS2581668 | 18 | 71142305 | 71.142305 | 2.5905 | 0.1075 |
| RS2581669 | 18 | 71142430 | 71.142430 | 0.6224 | 0.4302 |
| RS4891260 | 18 | 71144371 | 71.144371 | 0.5703 | 0.4501 |
| RS12962247 | 18 | 71150069 | 71.150069 | 0.8279 | 0.3629 |
| RS1866734 | 18 | 71151838 | 71.151838 | 0.9928 | 0.3191 |
| RS2581655 | 18 | 71152131 | 71.152131 | 1.6427 | 0.2000 |
| RS2639991 | 18 | 71152320 | 71.152320 | 2.2722 | 0.1317 |
| RS7230870 | 18 | 71158396 | 71.158396 | 0.9668 | 0.3255 |
| RS4891058 | 18 | 71165785 | 71.165785 | 0.0596 | 0.8071 |
| RS7407221 | 18 | 71168570 | 71.168570 | 2.1258 | 0.1448 |
| RS6566077 | 18 | 71171534 | 71.171534 | 1.9967 | 0.1576 |
| RS6566078 | 18 | 71171843 | 71.171843 | 0.1256 | 0.7230 |
| RS10514165 | 18 | 71173399 | 71.173399 | 5.7294 | 0.0167 |
| RS17192737 | 18 | 71174460 | 71.174460 | 1.1214 | 0.2896 |
| RS9965347 | 18 | 71176971 | 71.176971 | 1.0212 | 0.3122 |
| RS2222 | 18 | 71179492 | 71.179492 | 0.0813 | 0.7755 |
| **TSHZ2**  **PROBE** | **CHROM** | **START_LOCATION** | **MBp, Location** | **ARMIT_TREND** | **p-value** |
| RS6097158 | 20 | 50974873 | 50.974873 | 1.0268 | 0.3109 |
| RS732806 | 20 | 50983098 | 50.983098 | 0.4059 | 0.5241 |
| RS11905478 | 20 | 50989442 | 50.989442 | 4.5440 | 0.0330 |
| RS6097169 | 20 | 50990568 | 50.990568 | 0.0012 | 0.9729 |
| RS6013574 | 20 | 50996467 | 50.996467 | 0.8173 | 0.3660 |
| RS6097170 | 20 | 51002357 | 51.002357 | 1.2501 | 0.2635 |
| RS10470029 | 20 | 51005209 | 51.005209 | 0.1283 | 0.7202 |
| RS11700089 | 20 | 51008817 | 51.008817 | 3.2587 | 0.0710 |
| RS2093151 | 20 | 51010207 | 51.010207 | 0.1326 | 0.7157 |
| RS6068407 | 20 | 51013811 | 51.013811 | 0.3499 | 0.5542 |
| RS6097174 | 20 | 51014654 | 51.014654 | 2.0030 | 0.1570 |
| RS10485813 | 20 | 51017390 | 51.017390 | 4.6630 | 0.0308 |
| RS6013586 | 20 | 51023941 | 51.023941 | 0.0980 | 0.7542 |
| RS6022196 | 20 | 51031721 | 51.031721 | 0.2802 | 0.5966 |
| RS6022197 | 20 | 51032231 | 51.032231 | 1.1817 | 0.2770 |
| RS6022204 | 20 | 51052745 | 51.052745 | 5.5021 | 0.0190 |
| RS6013594 | 20 | 51052942 | 51.052942 | 0.0396 | 0.8423 |
| RS2252098 | 20 | 51053708 | 51.053708 | 0.1753 | 0.6754 |
| RS2252225 | 20 | 51055492 | 51.055492 | 0.2689 | 0.6041 |
| RS6022207 | 20 | 51062271 | 51.062271 | 1.3134 | 0.2518 |
| RS2741369 | 20 | 51064590 | 51.064590 | 0.0032 | 0.9548 |
| RS6097189 | 20 | 51067371 | 51.067371 | 0.9374 | 0.3330 |
| RS1950889 | 20 | 51068300 | 51.068300 | 2.9081 | 0.0881 |
| RS6022215 | 20 | 51070036 | 51.070036 | 0.0527 | 0.8184 |
| RS2741367 | 20 | 51070466 | 51.070466 | 2.3242 | 0.1274 |
| RS1022597 | 20 | 51072796 | 51.072796 | 1.3397 | 0.2471 |
| RS6126728 | 20 | 51076536 | 51.076536 | 1.9894 | 0.1584 |
| RS11906081 | 20 | 51077167 | 51.077167 | 0.2389 | 0.6250 |
| RS6022231 | 20 | 51080206 | 51.080206 | 0.4057 | 0.5242 |
| RS6123243 | 20 | 51082588 | 51.082588 | 0.1425 | 0.7058 |
| RS964886 | 20 | 51083355 | 51.083355 | 1.9778 | 0.1596 |
| RS17328948 | 20 | 51085574 | 51.085574 | 0.9321 | 0.3343 |
| RS10485815 | 20 | 51096895 | 51.096895 | 0.1559 | 0.6929 |
| RS6091627 | 20 | 51103779 | 51.103779 | 0.0013 | 0.9708 |
| RS6068445 | 20 | 51108814 | 51.108814 | 1.0889 | 0.2967 |
| RS6126742 | 20 | 51110823 | 51.110823 | 0.9172 | 0.3382 |
| RS2904295 | 20 | 51111377 | 51.111377 | 0.1815 | 0.6701 |
| RS1315869 | 20 | 51119526 | 51.119526 | 0.1937 | 0.6598 |
| RS1936966 | 20 | 51125186 | 51.125186 | 3.5185 | 0.0607 |
| RS1293442 | 20 | 51129218 | 51.129218 | 2.2626 | 0.1325 |
| RS910382 | 20 | 51132596 | 51.132596 | 0.3843 | 0.5353 |
| RS4811418 | 20 | 51133163 | 51.133163 | 2.1240 | 0.1450 |
| RS1936963 | 20 | 51133224 | 51.133224 | 1.1089 | 0.2923 |
| RS1936962 | 20 | 51138511 | 51.138511 | 1.1222 | 0.2894 |
| RS1293448 | 20 | 51141011 | 51.141011 | 3.7571 | 0.0526 |
| RS1317352 | 20 | 51147482 | 51.147482 | 2.3575 | 0.1247 |
| RS17390234 | 20 | 51148917 | 51.148917 | 2.0420 | 0.1530 |
| RS2801003 | 20 | 51148982 | 51.148982 | 1.7917 | 0.1807 |
| RS6097233 | 20 | 51150583 | 51.150583 | 1.2886 | 0.2563 |
| RS2800995 | 20 | 51153258 | 51.153258 | 2.7865 | 0.0951 |
| RS16997525 | 20 | 51153829 | 51.153829 | 3.5510 | 0.0595 |
| RS6022281 | 20 | 51155088 | 51.155088 | 0.0754 | 0.7836 |
| RS6126755 | 20 | 51156030 | 51.156030 | 0.0182 | 0.8926 |
| RS880430 | 20 | 51161023 | 51.161023 | 1.4515 | 0.2283 |
| RS8117683 | 20 | 51164598 | 51.164598 | 6.0583 | 0.0138 |
| RS1293427 | 20 | 51171008 | 51.171008 | 2.9219 | 0.0874 |
| RS1293425 | 20 | 51173585 | 51.173585 | 0.1534 | 0.6953 |
| RS2107325 | 20 | 51175023 | 51.175023 | 2.3974 | 0.1215 |
| RS11697971 | 20 | 51178193 | 51.178193 | 2.3024 | 0.1292 |
| RS1293421 | 20 | 51180675 | 51.180675 | 0.0386 | 0.8443 |
| RS16997581 | 20 | 51184527 | 51.184527 | 0.3562 | 0.5506 |
| RS2107323 | 20 | 51184613 | 51.184613 | 1.9425 | 0.1634 |
| RS16997592 | 20 | 51187544 | 51.187544 | 2.2577 | 0.1330 |
| RS6091638 | 20 | 51188672 | 51.188672 | 4.0642 | 0.0438 |
| RS1293417 | 20 | 51192328 | 51.192328 | 0.6192 | 0.4314 |
| RS1293413 | 20 | 51194691 | 51.194691 | 0.8571 | 0.3545 |
| RS10485451 | 20 | 51197172 | 51.197172 | 0.5973 | 0.4396 |
| RS1293412 | 20 | 51202023 | 51.202023 | 2.3086 | 0.1287 |
| RS6123255 | 20 | 51204098 | 51.204098 | 2.4978 | 0.1140 |
| RS8124782 | 20 | 51209247 | 51.209247 | 1.6474 | 0.1993 |
| RS1293405 | 20 | 51210315 | 51.210315 | 0.8988 | 0.3431 |
| RS973667 | 20 | 51213734 | 51.213734 | 0.7792 | 0.3774 |
| RS11086417 | 20 | 51214736 | 51.214736 | 1.5733 | 0.2097 |
| RS1293395 | 20 | 51218109 | 51.218109 | 0.5574 | 0.4553 |
| RS2801008 | 20 | 51222125 | 51.222125 | 0.8356 | 0.3607 |
| RS6123256 | 20 | 51223222 | 51.223222 | 0.7667 | 0.3812 |
| RS6097274 | 20 | 51225729 | 51.225729 | 0.8547 | 0.3552 |
| RS2801009 | 20 | 51226443 | 51.226443 | 0.6365 | 0.4250 |
| RS6022321 | 20 | 51226444 | 51.226444 | 0.2032 | 0.6521 |
| RS1293387 | 20 | 51230177 | 51.230177 | 1.3928 | 0.2379 |
| RS6068477 | 20 | 51233994 | 51.233994 | 0.0799 | 0.7774 |
| RS757365 | 20 | 51238361 | 51.238361 | 1.5397 | 0.2147 |
| RS1293381 | 20 | 51242306 | 51.242306 | 3.0492 | 0.0808 |
| RS2426460 | 20 | 51243518 | 51.243518 | 3.5889 | 0.0582 |
| RS1296358 | 20 | 51244793 | 51.244793 | 3.4402 | 0.0636 |
| RS1108344 | 20 | 51258519 | 51.258519 | 0.9612 | 0.3269 |
| RS1296032 | 20 | 51262620 | 51.262620 | 0.0990 | 0.7530 |
| RS1297517 | 20 | 51262938 | 51.262938 | 0.4098 | 0.5221 |
| RS6022333 | 20 | 51263442 | 51.263442 | 0.1580 | 0.6910 |
| RS1293363 | 20 | 51263767 | 51.263767 | 0.0030 | 0.9566 |
| RS6013649 | 20 | 51267431 | 51.267431 | 0.5461 | 0.4599 |
| RS2107332 | 20 | 51268772 | 51.268772 | 0.1874 | 0.6651 |
| RS12625311 | 20 | 51270200 | 51.270200 | 1.3933 | 0.2378 |
| RS6022341 | 20 | 51272472 | 51.272472 | 1.2161 | 0.2701 |
| RS16997769 | 20 | 51277141 | 51.277141 | 2.1263 | 0.1448 |
| RS6022348 | 20 | 51281479 | 51.281479 | 0.3889 | 0.5329 |
| RS2426472 | 20 | 51290219 | 51.290219 | 0.0049 | 0.9443 |
| RS6022352 | 20 | 51291600 | 51.291600 | 0.8630 | 0.3529 |
| RS916954 | 20 | 51292709 | 51.292709 | 0.0521 | 0.8195 |
| RS2426474 | 20 | 51295365 | 51.295365 | 1.4987 | 0.2209 |
| RS16997806 | 20 | 51301263 | 51.301263 | 0.3760 | 0.5397 |
| RS7345895 | 20 | 51302533 | 51.302533 | 0.8380 | 0.3600 |
| RS739870 | 20 | 51304256 | 51.304256 | 0.2352 | 0.6277 |
| RS6091653 | 20 | 51307119 | 51.307119 | 0.2311 | 0.6307 |
| RS1355597 | 20 | 51308176 | 51.308176 | 0.3775 | 0.5389 |
| RS6022360 | 20 | 51313268 | 51.313268 | 0.0072 | 0.9324 |
| RS6126802 | 20 | 51314446 | 51.314446 | 0.8502 | 0.3565 |
| RS6091655 | 20 | 51317983 | 51.317983 | 0.5182 | 0.4716 |
| RS6126805 | 20 | 51321064 | 51.321064 | 0.7988 | 0.3714 |
| RS1877430 | 20 | 51321529 | 51.321529 | 0.1019 | 0.7496 |
| RS6097326 | 20 | 51324797 | 51.324797 | 1.0313 | 0.3099 |
| RS1543080 | 20 | 51330522 | 51.330522 | 0.1941 | 0.6595 |
| RS6097334 | 20 | 51332771 | 51.332771 | 1.2215 | 0.2691 |
| RS2668791 | 20 | 51334138 | 51.334138 | 0.0830 | 0.7733 |
| RS2137299 | 20 | 51335096 | 51.335096 | 0.4683 | 0.4938 |
| RS169271 | 20 | 51342873 | 51.342873 | 0.1417 | 0.7066 |
| RS6013656 | 20 | 51344606 | 51.344606 | 0.6709 | 0.4127 |
| RS8125417 | 20 | 51345189 | 51.345189 | 1.1354 | 0.2866 |
| RS932533 | 20 | 51346353 | 51.346353 | 0.2627 | 0.6082 |
| RS200633 | 20 | 51346403 | 51.346403 | 0.1429 | 0.7054 |
| RS16997896 | 20 | 51346815 | 51.346815 | 0.9266 | 0.3358 |
| RS4811423 | 20 | 51348131 | 51.348131 | 0.8929 | 0.3447 |
| RS6512878 | 20 | 51348486 | 51.348486 | 1.2671 | 0.2603 |
| RS6097345 | 20 | 51351578 | 51.351578 | 0.0001 | 0.9931 |
| RS13039689 | 20 | 51355478 | 51.355478 | 0.3408 | 0.5594 |
| RS1996017 | 20 | 51356045 | 51.356045 | 0.0189 | 0.8907 |
| RS8120425 | 20 | 51357904 | 51.357904 | 1.0190 | 0.3128 |
| RS1355598 | 20 | 51358916 | 51.358916 | 0.0147 | 0.9036 |
| RS6097354 | 20 | 51359242 | 51.359242 | 0.3370 | 0.5615 |
| RS200597 | 20 | 51361318 | 51.361318 | 3.4438 | 0.0635 |
| RS6063929 | 20 | 51362115 | 51.362115 | 3.1360 | 0.0766 |
| RS6091661 | 20 | 51364361 | 51.364361 | 1.7479 | 0.1861 |
| RS200655 | 20 | 51365788 | 51.365788 | 4.2200 | 0.0399 |
| RS200654 | 20 | 51366120 | 51.366120 | 0.0150 | 0.9024 |
| RS200646 | 20 | 51371913 | 51.371913 | 0.0656 | 0.7978 |
| RS6068509 | 20 | 51376306 | 51.376306 | 4.2407 | 0.0395 |
| RS169273 | 20 | 51377233 | 51.377233 | 0.1706 | 0.6796 |
| RS200641 | 20 | 51378068 | 51.378068 | 0.3594 | 0.5488 |
| RS13044509 | 20 | 51378919 | 51.378919 | 0.3075 | 0.5792 |
| RS200607 | 20 | 51381063 | 51.381063 | 0.5686 | 0.4508 |
| RS7266390 | 20 | 51381809 | 51.381809 | 0.0610 | 0.8050 |
| RS200603 | 20 | 51385908 | 51.385908 | 0.3547 | 0.5515 |
| RS200599 | 20 | 51387409 | 51.387409 | 0.0024 | 0.9609 |
| RS2741356 | 20 | 51387954 | 51.387954 | 1.0095 | 0.3150 |
| RS200609 | 20 | 51389657 | 51.389657 | 0.0147 | 0.9036 |
| RS6022405 | 20 | 51392738 | 51.392738 | 1.4190 | 0.2336 |
| RS200623 | 20 | 51394054 | 51.394054 | 0.7897 | 0.3742 |
| RS200624 | 20 | 51394967 | 51.394967 | 0.3731 | 0.5413 |
| RS169269 | 20 | 51396655 | 51.396655 | 1.2287 | 0.2677 |
| RS169270 | 20 | 51399798 | 51.399798 | 0.2617 | 0.6090 |
| RS6013667 | 20 | 51400261 | 51.400261 | 2.0283 | 0.1544 |
| RS200629 | 20 | 51402847 | 51.402847 | 0.0707 | 0.7904 |
| RS200631 | 20 | 51404559 | 51.404559 | 0.8323 | 0.3616 |
| RS16997981 | 20 | 51408698 | 51.408698 | 0.1726 | 0.6778 |
| RS2904359 | 20 | 51410644 | 51.410644 | 0.3632 | 0.5467 |
| RS465580 | 20 | 51411354 | 51.411354 | 0.1109 | 0.7391 |
| RS156598 | 20 | 51416423 | 51.416423 | 0.4400 | 0.5071 |
| RS6063931 | 20 | 51418597 | 51.418597 | 0.8081 | 0.3687 |
| RS465880 | 20 | 51422033 | 51.422033 | 2.5195 | 0.1124 |
| RS10485447 | 20 | 51423786 | 51.423786 | 0.4975 | 0.4806 |
| RS156617 | 20 | 51426171 | 51.426171 | 0.5519 | 0.4575 |
| RS156618 | 20 | 51426981 | 51.426981 | 0.0797 | 0.7777 |
| RS156627 | 20 | 51436702 | 51.436702 | 2.0908 | 0.1482 |
| RS156628 | 20 | 51436820 | 51.436820 | 0.2494 | 0.6175 |
| RS16984628 | 20 | 51442519 | 51.442519 | 0.9840 | 0.3212 |
| RS17443093 | 20 | 51442609 | 51.442609 | 1.7610 | 0.1845 |
| RS156601 | 20 | 51443548 | 51.443548 | 0.3845 | 0.5352 |
| RS468211 | 20 | 51450645 | 51.450645 | 1.1682 | 0.2798 |
| RS156604 | 20 | 51450933 | 51.450933 | 4.2194 | 0.0400 |
| RS4811429 | 20 | 51458409 | 51.458409 | 0.4848 | 0.4862 |
| RS10485445 | 20 | 51466201 | 51.466201 | 0.3114 | 0.5768 |
| RS6063942 | 20 | 51471354 | 51.471354 | 1.4195 | 0.2335 |
| RS6123281 | 20 | 51478713 | 51.478713 | 2.8474 | 0.0915 |
| RS6022462 | 20 | 51484172 | 51.484172 | 1.0547 | 0.3044 |
| RS6013688 | 20 | 51493399 | 51.493399 | 0.9928 | 0.3191 |
| RS7360757 | 20 | 51493474 | 51.493474 | 0.0022 | 0.9629 |
| RS6063947 | 20 | 51501548 | 51.501548 | 0.0899 | 0.7643 |
| RS6068555 | 20 | 51516261 | 51.516261 | 0.1704 | 0.6797 |
| RS2741373 | 20 | 51539379 | 51.539379 | 0.2218 | 0.6377 |
| RS2275005 | 20 | 51541872 | 51.541872 | 1.2364 | 0.2662 |
| RS1999602 | 20 | 51545423 | 51.545423 | 0.1918 | 0.6614 |
| RS6097458 | 20 | 51548300 | 51.548300 | 1.4188 | 0.2336 |
| RS8117157 | 20 | 51566077 | 51.566077 | 0.0169 | 0.8966 |
| RS6022540 | 20 | 51568107 | 51.568107 | 0.5528 | 0.4572 |
| RS2741383 | 20 | 51570630 | 51.570630 | 0.0300 | 0.8624 |
| RS2766641 | 20 | 51572325 | 51.572325 | 0.0051 | 0.9432 |
| RS2904362 | 20 | 51580068 | 51.580068 | 0.1601 | 0.6890 |
| RS2741390 | 20 | 51584592 | 51.584592 | 0.0962 | 0.7564 |
| RS756059 | 20 | 51586230 | 51.586230 | 0.1267 | 0.7219 |
| **TSHZ3**  **PROBE** | **CHROM** | **START_LOCATION** | **MBp, Location** | **ARMIT_TREND** | **p-value** |
| RS1948557 | 19 | 36410329 | 36.410329 | 0.1039 | 0.7472 |
| RS4805660 | 19 | 36443099 | 36.443099 | 0.6441 | 0.4222 |
| RS4805661 | 19 | 36450115 | 36.450115 | 0.0020 | 0.9647 |
| RS1496625 | 19 | 36452469 | 36.452469 | 1.6694 | 0.1963 |
| RS8104214 | 19 | 36454744 | 36.454744 | 0.0307 | 0.8609 |
| RS10423293 | 19 | 36457119 | 36.457119 | 1.6871 | 0.1940 |
| RS3745784 | 19 | 36460256 | 36.460256 | 0.1058 | 0.7450 |
| RS12461253 | 19 | 36461603 | 36.461603 | 0.2562 | 0.6128 |
| RS10425374 | 19 | 36466506 | 36.466506 | 0.3819 | 0.5366 |
| RS13345671 | 19 | 36467410 | 36.467410 | 1.5962 | 0.2064 |
| ***RS4805666*** | ***19*** | ***36469347*** | ***36.469347*** | ***7.7899*** | ***0.0053*** |
| RS4805667 | 19 | 36469505 | 36.469505 | 5.0638 | 0.0244 |
| RS10423093 | 19 | 36472721 | 36.472721 | 0.1116 | 0.7383 |
| RS17496703 | 19 | 36476435 | 36.476435 | 1.1572 | 0.2820 |
| RS7251762 | 19 | 36479800 | 36.479800 | 2.8672 | 0.0904 |
| ***RS7255674*** | ***19*** | ***36480765*** | ***36.480765*** | ***6.7266*** | ***0.0095*** |
| RS11883254 | 19 | 36482162 | 36.482162 | 0.1452 | 0.7032 |
| RS16965432 | 19 | 36488129 | 36.488129 | 1.9755 | 0.1599 |
| RS10411550 | 19 | 36489009 | 36.489009 | 0.0043 | 0.9477 |
| RS1078373 | 19 | 36492970 | 36.492970 | 0.0536 | 0.8170 |
| RS7253629 | 19 | 36493169 | 36.493169 | 0.5360 | 0.4641 |
| RS10412617 | 19 | 36494390 | 36.494390 | 0.4276 | 0.5132 |
